# The Burden of Nonmalignant Metabolic Dysfunction‐Associated Steatotic Liver Disease in the Western Pacific Region: A Systematic Analysis From 1990 to 2021

**DOI:** 10.1002/fsn3.70627

**Published:** 2025-07-16

**Authors:** Qichao Ge, Yuan Lin, Mingwang Wang, Jianwei Zhu, Qingqing Zhang, Junjun Wang, Yufei Yang, Hanjing Zhangdi, Yuecheng Guo, Shanjuan Wang, Lungen Lu

**Affiliations:** ^1^ Department of Gastroenterology Shanghai General Hospital, Shanghai Jiao Tong University School of Medicine Shanghai China; ^2^ Department of Gastroenterology Jiading District Central Hospital Affiliated Shanghai University of Medicine & Health Science Shanghai China; ^3^ Department of Gastroenterology The First Affiliated Hospital of Soochow University Suzhou Jiangsu China

**Keywords:** epidemiology, global burden of disease 2021, metabolic dysfunction‐associated steatotic liver disease, western pacific region

## Abstract

The burden of metabolic dysfunction‐associated steatotic liver disease (MASLD) is increasing, yet there is little understanding in the Western Pacific Region (WPR). This study aims to report the latest MASLD burden in the WPR from 1990 to 2021. This study obtained nonmalignant and total MASLD incidence, prevalence, mortality, disability‐adjusted life years (DALYs), years of life lost (YLLs), and years of healthy life lost due to disability (YLDs) from the Global Burden of Disease (GBD) database, and calculated age‐standardized ratios (ASRs) for each indicator. Estimated annual percentage changes (EAPCs) were calculated using a log‐transformed linear regression model. The age‐period‐cohort (APC) model was used to analyze regional MASLD‐related deaths; the Bayesian age‐period‐cohort (BAPC) model was used to estimate future trends from 2022 to 2050. The incidence, prevalence, deaths, and DALYs of nonmalignant MASLD in the WPR in 2021 are 12 million, 359.2 million, 10,195 cases, and 0.24 million years, respectively. The WPR has a high incidence of MASLD in younger age groups, especially in males, with the 15–19‐year‐old male group being 1215.82 per 100,000. Mongolia has the highest relative risk of deaths and DALYs, while Japan, Singapore, and China are comparatively low. The proportion of deaths and DALYs due to nonmalignant MASLD is increasing. Nonmalignant MASLD is projected to be the dominant component of resulting deaths and DALYs by 2050 in some countries. The WPR burden of MASLD is increasing, with a high prevalence in younger individuals, indicating the need for early initiation of targeted and effective MASLD prevention.

## Graphical abstract

The overall GBD descriptions of non‐malignant MASLD in the western pacific region. (A) Estimated annual percentage changes (EAPCs) in age standardized rates of incidence for non‐malignant MASLD by country, for the period 1990–2021. (B) EAPCs in age standardized rates of prevalence for non‐malignant MASLD by country, for the period 1990–2021. (C) EAPCs in age standardized rates of deaths for non‐malignant MASLD by country, for the period 1990–2021. (D) EAPCs in age standardized rates of disability adjusted life years (DALYs) for non‐malignant MASLD by country, for the period 1990–2021. (E) EAPCs in age standardized rates of years of healthy life lost due to disability (YLDs) for non‐malignant MASLD by country, for the period 1990–2021. (F) EAPCs in age standardized rates of years of life lost (YLLs) for non‐malignant MASLD by country, for the period 1990–2021.

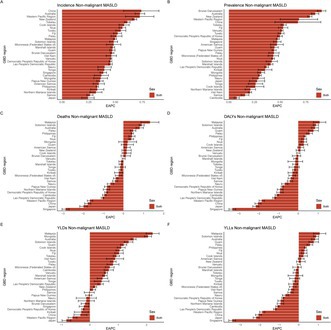

**Abbreviations**
APC: age‐period‐cohort
ARS: age‐standardized ratio
ASDR: age‐standardized death rate
ASIR: age‐standardized incidence rate
ASPR: age‐standardized prevalence rate
BAPC: Bayesian age‐period‐cohort
DALYs: disability adjusted life years
EAPC: estimated annual percentage change
GBD: global burden of disease
HBV: hepatitis B virus
HCC: hepatocellular carcinoma
HCV: hepatitis C virus
IHME: Institute for Health Metrics and Evaluation
MASLD: metabolic dysfunction‐associated steatotic liver disease
NAFLD: nonalcoholic fatty liver disease
NASH: nonalcoholic steatohepatitis
NCD: noncommunicable disease
SDG: sustainable development goal
SDI: sociodemographic index
UI: uncertainty interval
WHO: World Health Organization
YLDs: years of healthy life lost due to disability
YLLs: years of life lost

## 1 Introduction

Nonalcoholic fatty liver disease (NAFLD) is the hepatic manifestation of metabolic syndrome (MetS) and includes simple steatosis and nonalcoholic steatohepatitis (NASH). NAFLD has been usually linked to cardiovascular disease, metabolic syndrome, insulin resistance, and hepatocellular carcinoma (HCC) (Israelsen et al. 2024). The concept of NAFLD has been renamed as metabolic dysfunction‐associated steatotic liver disease (MASLD) recently (Rinella et al. 2023; Song et al. 2024). The prevalence of MASLD has escalated significantly in recent decades, currently impacting approximately 25% of the global population. It is anticipated to emerge as the predominant cause of advanced liver disease in the forthcoming decades (Younossi et al. 2017). MASLD is influenced by genetic factors, socioeconomic conditions, and metabolic disorders, resulting in a heterogeneous geographical distribution (Israelsen et al. 2024; Wong et al. 2023). A comprehensive analysis of regional disease burdens and trends can offer valuable insights for governments, healthcare providers, researchers, and other stakeholders. The Western Pacific region represents the most populous area within the World Health Organization's jurisdiction, characterized by significant geographical, cultural, ethnic, and socioeconomic diversity (Division of Healthy Environments and Populations (DHP) and WHO Western Pacific (WWP) 2023). Furthermore, this region is experiencing accelerating population aging, with more than 265 million people aged 65 years and above (the share of the over‐65 population of 13.8%) in 2021, which is linked to an increased burden of noncommunicable diseases and chronic conditions (Institute of Health Metrics and Evaluation (IHME) 2021; Kasai 2021). While the burden of MASLD has been widely reported in some countries such as China and Japan, how the specific burden of MASLD changed in each Western Pacific country and area has yet to be comprehensively reported using the most recent post COVID‐19 pandemic data, which would provide important evidence on investing in the prevention and control of MASLD and achieving the United Nations Sustainable Development Goals (SDG) target (Riazi et al. 2022; Tian et al. 2023; Chew et al. 2023).

The Global Burden of Disease Study (GBD) is an international research endeavor spearheaded by the Institute for Health Metrics and Evaluation at the University of Washington. The database provides death and disease rate assessments and analyzes risk factors' impact on health, making the GBD essential for global public health policy development and prioritization. Within the etiological framework defined by the GBD study, we consider the chronic progression of NAFL, NASH, liver fibrosis, and cirrhosis as the nonmalignant components of MASLD, while the development of HCC resulting from MASLD is regarded as malignant. In this study, nonmalignant MASLD coincides with NAFLD including cirrhosis in the GBD database. MASLD progression can be considered essentially linear, with NASH occurring in approximately 20% of patients and accelerating the process (Samy et al. 2024). Malignant MASLD predominantly progresses from cirrhosis, with an estimated 20%–50% of cases potentially advancing directly to HCC, contingent upon the presence of NASH (Hagström et al. 2024; Ioannou 2021). Conversely, the influence of MASLD on the development of extrahepatic malignancies may be underestimated. The incidence of extrahepatic tumors in individuals with MASLD has been estimated to be eight times higher than that of HCC and appears to be independent of the fibrosis stage (Thomas et al. 2024). Consequently, we propose that nonmalignant MASLD should be a primary focus in the management of the overall MASLD spectrum.

This study aims to systematically analyze overall and nonmalignant MASLD data from 1990 to 2021 in the Western Pacific region, with the objective of reporting the most recent disease burden. Furthermore, this study highlights differences between nonmalignant and total MASLD to enhance understanding of management benefits.

## 2 Methods

### 2.1 Overview

For this study, we performed secondary analyses utilizing newly released and publicly accessible data from the Global Burden of Disease (GBD) 2021. The GBD study offers iterative and continuous estimates of disease burdens, employing the most reliable data sources to assess global health loss. The most updated GBD 2021 utilized 5086 data sources to estimate age‐sex‐location‐year specific mortality and years of life lost (YLLs) for 288 causes of death in 204 countries and territories between 1990 and 2021, while 100,983 data sources were synthesized to produce estimates of DALYs for 371 diseases and injuries by age (25 age groups from birth to 95 years and older), sex (male, female, and both sexes combined), location (204 countriesand territories), and year (annually from 1990 to 2021) (Naghavi et al. 2024; Schumacher et al. 2024). All GBD 2021 health estimates are available at https://vizhub.healthdata.org/gbd‐results/.

To thoroughly examine the disease burden associated with total MASLD and nonmalignant MASLD in the Western Pacific region, our study concentrated on 31 countries and territories (hereafter “countries” for simplicity) in the WHO Western Pacific region, including American Samoa, Australia, Brunei Darussalam, Cambodia, China, the Cook Islands, Fiji, Guam, Japan, Kiribati, Laos, Malaysia, the Marshall Islands, the Federated States of Micronesia, Mongolia, Nauru, New Zealand, Niue, the Northern Mariana Islands, Palau, Papua New Guinea, the Philippines, the Republic of Korea, Samoa, Singapore, the Solomon Islands, Tokelau, Tonga, Tuvalu, Vanuatu, and Viet Nam. All rates are reported per 100,000 population, following epidemiological conventions.

### 2.2 Data Sources and Definition of Nonmalignant MASLD

All data for this study were obtained by searching the Global Health Data exchange with the GBD Results tool on the Institute for Health Metrics and Evaluation (IHME) website (Institute of Health Metrics and Evaluation (IHME) 2021). The primary data retrieval strategy is described as follows. This investigation used “Total burden related to MASLD cause” and “Nonalcoholic fatty liver disease including cirrhosis” set in the GBD study, with the latter excludes hepatocellular carcinoma conditions. The definition of nonmalignant MASLD coincided with NAFLD including cirrhosis in the GBD database. Incidence, prevalence, deaths, disability adjusted life years (DALYs), years of healthy life lost due to disability (YLDs), and years of life lost (YLLs) attributable to these two causes were collected for 31 countries in the Western Pacific region between 1990 and 2021.

### 2.3 Statistical Analysis

Incidence, prevalence, deaths, DALYs, YLDs, and YLLs were used to describe the absolute burden of nonmalignant and overall MASLD in 31 countries in the Western Pacific, with definitions consistent with the GBD study. The algorithm for DALYs is as follows:

$$\begin{array}{c} \text{DALYs}=\text{YLDs}+\text{YLLs}=\text{prevalence of the condition}\\ \;\times \text{disability weights}+\left(\text{life expectancy}–\text{death}\;\mathrm{age}\right) \end{array}$$

MASLD disability weights were determined by the GBD study through surveys based on pair‐wise comparison method. National data obtained directly from the GBD database, while the regional results were obtained from our secondary estimates. Age‐standardized ratios (ASRs) were calculated by direct standardization method using the age structure reported in GBD 2021 and were used to describe the relative burden of nonmalignant and total MASLD across countries and regions. In addition, we calculated the estimated annual percentage changes (EAPCs) for ARS over the period 1990–2021 through log‐transformed linear regression model, which were used to characterize the trends in each indicator.

Uncertainty intervals (UIs) characterized the uncertainty of the estimates by simulating 1000 random draws from the posterior distribution at each step of the modeling process and taking 95% (25th–975th) as the final result. All data analyses and visualizations were performed through the R software (version 4.3.2) and Stata/MP 17.0 (StataCorp LLC), and all R packages used can be accessed on the relevant database (https://rdrr.io).

### 2.4 Age‐Period‐Cohort Analysis

Age‐Period‐Cohort (APC) analysis is applied to describe effects along 3 dimensions: specific age stage, specific period for all age groups, and the same birth cohort subject to time‐ and experience‐related effects. We employed Age‐Period‐Cohort (APC) models to analyze deaths attributable to nonmalignant and overall MASLD. Due to the absence of data on MASLD‐related deaths for individuals aged 0–14 years, our study focused on an age cohort comprising 16 groups, each spanning 5‐year intervals, covering ages 15–94 years, along with an additional group for individuals aged 95 years and above. Correspondingly, the period cohort was structured into six 5‐year intervals from 1992 to 2021. Consequently, 22 partially overlapping 5‐year birth cohorts, ranging from 1897–1901 to 2002–2006, were utilized for the analysis.

### 2.5 Bayesian Age‐Period‐Cohort Estimate

China, Japan, Viet Nam, and the Philippines were selected for Bayesian Age‐period‐cohort (BAPC) predictive analyses based on their population sizes, Social Development Index (SDI) rankings, and previously determined EAPCs in incidence rates. In 2021, these countries ranked among the top four in terms of population within the Western Pacific region, collectively accounting for over 90% of the total population (Schumacher et al. 2024). Additionally, they exhibit a balanced distribution of social development levels and EAPCs of incidence. The R packages “BAPC” (version 0.0.36) and “INLA” (version 23.09.09) were employed to forecast disease burden, utilizing fully Bayesian inference through integrated nested Laplace approximations, which enhances accuracy in complex models and long‐term projections. Utilizing GBD data from 1990 to 2021, we projected indicators such as incidence and prevalence for total and nonmalignant MASLD up to the year 2050.

## 3 Results

### 3.1 Burden of Total MASLD in the Western Pacific Region

Based on data from the GBD 2021 study, the total MASLD incidence, prevalence, deaths, and DALYs in the Western Pacific region in 2021 were estimated to be 12.0 million (95% UI: 8.2–16.7), 359.2 million (95% UI: 278.4–451.6), 25,278 (95% UI: 17847–34611), and 600,447 (95% UI: 423839–829044), respectively. Between 1990 and 2021, there has been a gradual increase in the number of patients involved, but the trend of severe outcomes is improving, with EAPCs of 0.73 (95% UI: 0.59–0.86), 0.68 (95% UI: 0.5–0.87), −0.78 (95% UI: −0.84 to −0.71), and −1.07 (95% UI: −1.15 to −0.98) for the above indicators (Table 1, and Tables S1–S3, S7–S9).

**TABLE 1:** Age‐standardized death rate and estimated annual percentage change of total and nonmalignant MASLD in the Western Pacific region in 1990–2021.

Location	Total MASLD			Nonmalignant MASLD		
	1990 ASDR per 100,000 (95% UI)	2021 ASDR per 100,000 (95% UI)	EAPC of ASDR (95% UI)	1990 ASDR per 100,000 (95% UI)	2021 ASDR per 100,000 (95% UI)	EAPC of ASDR (95% UI)
Western Pacific Region	1.65 (1.15, 2.36)	1.27 (0.9, 1.75)	−0.78 (−0.84, −0.71)	0.87 (0.46, 1.49)	0.52 (0.28, 0.87)	−1.66 (−1.79, −1.52)
High SDI (> 0.81)						
Democratic People's Republic of Korea	1.43 (0.73, 2.62)	1.12 (0.59, 1.95)	−0.81 (−0.88, −0.75)	0.64 (0.27, 1.33)	0.48 (0.2, 1)	−0.86 (−0.93, −0.78)
Japan	1.88 (1.31, 2.69)	1.07 (0.72, 1.53)	−1.99 (−2.22, −1.77)	1.2 (0.67, 1.99)	0.62 (0.33, 1.04)	−1.98 (−2.16, −1.79)
Singapore	1.17 (0.72, 1.79)	0.72 (0.45, 1.11)	−1.53 (−1.81, −1.24)	0.63 (0.31, 1.13)	0.26 (0.14, 0.46)	−2.87 (−3.04, −2.69)
New Zealand	0.86 (0.58, 1.22)	1.27 (0.93, 1.64)	1.17 (1.02, 1.32)	0.62 (0.36, 0.97)	0.66 (0.4, 0.95)	0.16 (−0.07, 0.39)
Australia	1.14 (0.7, 1.77)	1.87 (1.27, 2.67)	1.86 (1.72, 2)	0.93 (0.52, 1.54)	1.08 (0.64, 1.65)	0.81 (0.63, 0.99)
High‐middle SDI (0.70–0.81)						
Brunei Darussalam	1.88 (1.09, 3.14)	1.59 (0.92, 2.54)	−0.53 (−0.64, −0.41)	0.65 (0.3, 1.23)	0.59 (0.27, 1.11)	0.12 (−0.12, 0.35)
Guam	2.56 (1.46, 4.33)	2.71 (1.59, 4.34)	0.73 (0.48, 0.99)	2.09 (1.03, 3.85)	1.85 (0.9, 3.37)	0.2 (−0.11, 0.5)
Cook Islands	1.77 (0.91, 3.13)	2.05 (1.06, 3.6)	0.37 (0.14, 0.6)	0.15 (0.06, 0.3)	0.18 (0.07, 0.36)	0.16 (−0.06, 0.38)
Northern Mariana Islands	4.72 (2.61, 8.13)	4.65 (2.73, 7.44)	−0.52 (−0.66, −0.37)	3.79 (1.84, 7.03)	3.38 (1.65, 5.95)	−0.84 (−1, −0.68)
Palau	2.29 (1.24, 4.01)	2.82 (1.49, 4.88)	0.49 (0.26, 0.72)	1.51 (0.65, 3)	1.83 (0.77, 3.63)	0.48 (0.34, 0.62)
Malaysia	1.22 (0.72, 1.95)	2.11 (1.26, 3.36)	1.49 (1.02, 1.97)	0.63 (0.31, 1.17)	1.16 (0.54, 2.17)	1.32 (0.87, 1.76)
vNiue	1.96 (1.07, 3.41)	2.56 (1.32, 4.37)	0.61 (0.46, 0.76)	1.35 (0.61, 2.65)	1.67 (0.7, 3.27)	0.39 (0.28, 0.51)
American Samoa	2.54 (1.43, 4.28)	3.41 (2, 5.47)	0.74 (0.55, 0.93)	1.76 (0.82, 3.33)	2.07 (0.99, 3.72)	0.18 (0.03, 0.34)
China	1.46 (1.01, 2.09)	1.15 (0.8, 1.59)	−0.58 (−0.72, −0.45)	0.75 (0.39, 1.31)	0.44 (0.23, 0.75)	−1.77 (−1.92, −1.62)
Low to middle SDI (< 0.70)						
Tokelau	1.77 (0.87, 3.33)	1.96 (1.06, 3.33)	0.03 (−0.1, 0.17)	1.21 (0.51, 2.47)	1.24 (0.56, 2.37)	−0.12 (−0.22, −0.03)
Fiji	1.22 (0.66, 2.18)	1.65 (0.91, 2.71)	0.85 (0.78, 0.92)	0.67 (0.29, 1.35)	0.84 (0.37, 1.62)	0.42 (0.27, 0.58)
Philippines	1.37 (0.82, 2.31)	1.49 (1.04, 2.11)	0.32 (0.27, 0.36)	0.64 (0.28, 1.32)	0.71 (0.37, 1.22)	0.44 (0.38, 0.51)
Viet Nam	2.38 (1.29, 4.19)	2.26 (1.26, 3.77)	−0.06 (−0.12, 0.01)	0.91 (0.37, 1.98)	0.81 (0.35, 1.57)	−0.38 (−0.49, −0.27)
Tonga	5.47 (3.06, 9.36)	6.16 (3.44, 10.14)	0.01 (−0.36, 0.39)	2.62 (1.19, 5.03)	2.75 (1.19, 5.34)	−0.16 (−0.42, 0.1)
Nauru	2.88 (1.51, 5.01)	2.58 (1.13, 4.77)	−0.83 (−1.04, −0.62)	2 (0.84, 3.99)	1.85 (0.62, 3.87)	−0.64 (−0.8, −0.49)
Mongolia	8.7 (5.31, 13.69)	12.12 (7.42, 19.09)	1.51 (1.09, 1.94)	4.13 (2.01, 7.54)	3.88 (1.9, 6.93)	0.21 (−0.23, 0.66)
Samoa	2.15 (1.16, 3.7)	2.09 (1.13, 3.5)	−0.39 (−0.53, −0.26)	1.38 (0.59, 2.75)	1.33 (0.6, 2.57)	−0.41 (−0.54, −0.28)
Micronesia (Federated States of)	2.38 (1.25, 4.28)	2.55 (1.34, 4.42)	0.01 (−0.22, 0.24)	1.74 (0.75, 3.5)	1.73 (0.74, 3.43)	−0.24 (−0.41, −0.07)
Tuvalu	2.04 (1.03, 3.79)	2.14 (1.18, 3.64)	0.04 (−0.03, 0.1)	1.43 (0.59, 2.94)	1.4 (0.64, 2.74)	−0.16 (−0.22, −0.1)
Marshall Islands	1.81 (0.85, 3.37)	1.95 (0.98, 3.52)	0.11 (0.01, 0.21)	1.39 (0.57, 2.76)	1.39 (0.57, 2.82)	−0.14 (−0.21, −0.06)
Kiribati	2.72 (1.45, 4.78)	2.81 (1.53, 4.8)	−0.16 (−0.42, 0.09)	1.78 (0.72, 3.61)	1.8 (0.79, 3.59)	−0.23 (−0.44, −0.02)
Lao People's Democratic Republic	2.34 (1.21, 4.25)	1.78 (0.98, 3.03)	−1.07 (−1.13, −1.01)	1.25 (0.49, 2.76)	0.95 (0.41, 1.93)	−1.04 (−1.1, −0.98)
Cambodia	3.66 (1.66, 7.85)	3.02 (1.58, 5.35)	−0.83 (−0.91, −0.74)	2.61 (1.01, 6.14)	2.09 (0.91, 4.26)	−0.95 (−1.04, −0.86)
Vanuatu	2.07 (0.87, 4.65)	2.19 (1.07, 4.2)	0.13 (0.05, 0.21)	1.5 (0.51, 3.64)	1.56 (0.6, 3.33)	0.01 (−0.06, 0.09)
Solomon Islands	1.9 (0.82, 3.99)	2.26 (1.19, 4.02)	0.5 (0.34, 0.66)	1.27 (0.5, 2.72)	1.69 (0.78, 3.26)	0.91 (0.78, 1.03)
Papua New Guinea	0.78 (0.34, 1.78)	0.61 (0.29, 1.26)	−1.07 (−1.17, −0.98)	0.36 (0.14, 0.75)	0.3 (0.13, 0.6)	−0.81 (−0.94, −0.68)

*Note:* The SDI of a region or country is related to the development of each period, SDI values and grading for each country are determined by the GBD database.

Abbreviations: ASDR, age‐standardized death rate; EAPC, estimated annual percentage change; MASLD, metabolic dysfunction‐associated steatotic liver disease; SDI, sociodemographic index.

**FIGURE 1:**
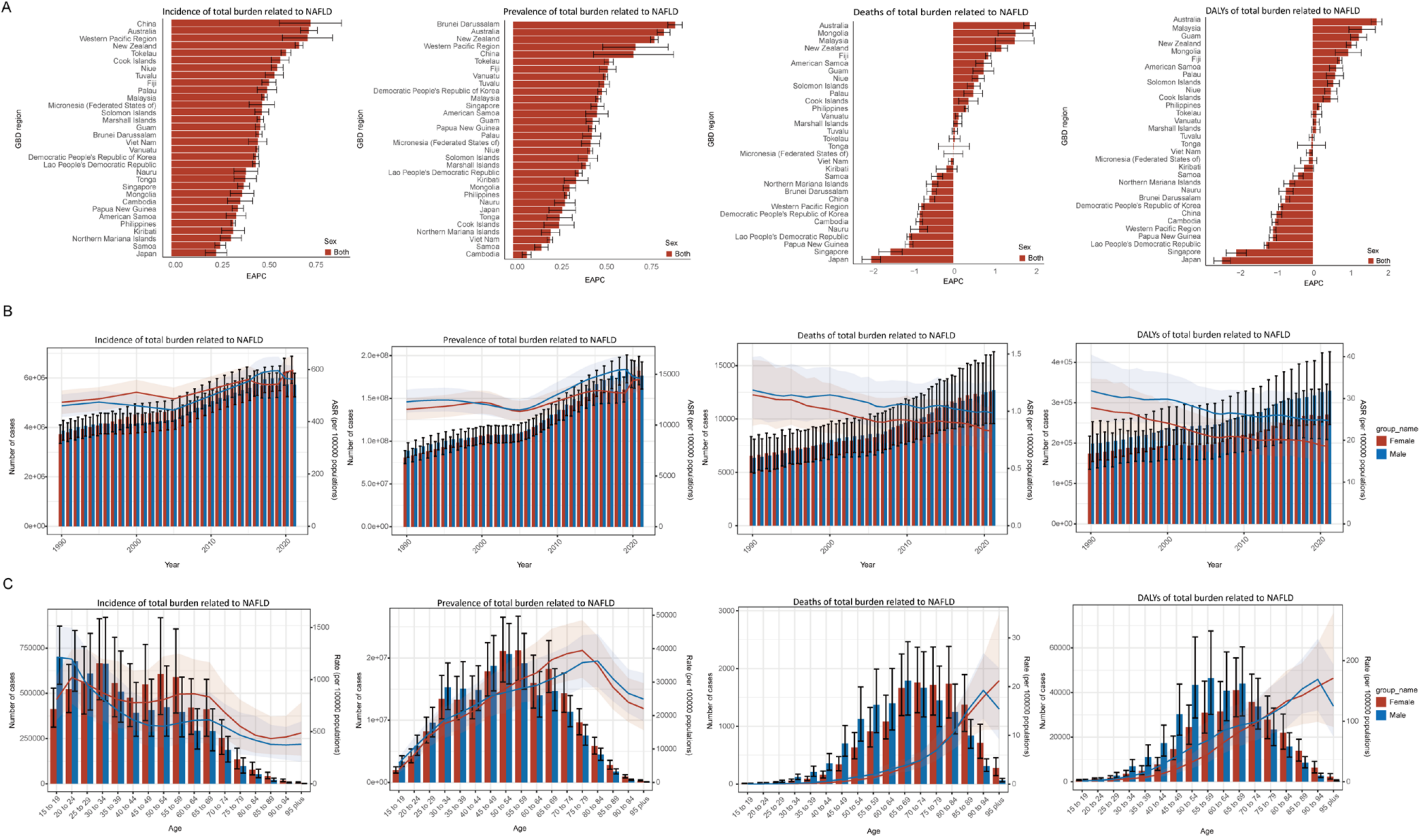
Characterization of total MASLD burden in the Western Pacific region in 1990–2021. (A) Estimated annual percentage changes in age standardized rates of incidence, prevalence, deaths, and DALYs for total MASLD by country, for the period 1990–2021. An EAPC of less than 0 represents a decrease, while greater than 0 indicates an increase, and an uncertainty interval spanning 0 is considered a nonsignificant result. (B) Changes in incidence, prevalence, deaths, and DALYs of total MASLD by year in the Western Pacific between 1990 and 2021. Bar lengths represent values for the current year, and line positions indicate values after age‐standardization. (C) Changes in incidence, prevalence, deaths, and DALYs of total MASLD by age in the Western Pacific between 1990 and 2021. Bar lengths represent values for the current year, and line positions indicate values after age‐standardization. DALY, disability adjusted life year; EAPC, estimated annual percentage change; GBD, global burden of disease.

In the Western Pacific region, the temporal trends of the indicators were broadly similar for both sexes, with age‐standardized incidence and prevalence increasing while deaths and DALYs decreasing. Notably, the age‐standardized incidence rate (ASIR) for total MASLD across genders showed intriguing inflection points in 2015 and 2019, with theatrical changes after 2019, and thus changes at these points warrant critical consideration. ASRs of male deaths, DALYs, and YLLs are higher compared to female deaths; for example, 1.39 cases (95% UI: 0.94–1.96) per 100,000 vs. 1.16 (95% UI: 0.79–1.62) in 2021 (Figure 1B and Table S10). For age distribution, total MASLD incidence in females shows two peaks at ages 20–24 and 55–69, with ASIR at 1021.04 (95% UI: 768.32–1287.8) per 100,000 and 859.86 (95% UI: 570.2–1247.63), respectively (Table S11). There is an overall declining trend in male incidence, with a slight rebound at ages 55–69. To note, incidence in adolescent males is remarkably high, with an estimated ASIR of 1215.82 (95% UI: 955.28–1512.81) per 100,000 for ages 15–19 (Table S11). Deaths and DALYs continued to rise with close differences across genders, but in the > 90 years, women's risk continued to rise while men's shifted to decline (Figure 1C). ASDR tops out at 21.21 (95% UI: 11.15–34.69) per 100,000 for females aged over 95 and 19.23 (95% UI: 13.42–26.87) for males aged 90–94 (Table S11).

State MASLD burdens vary widely. ASIRs and age‐standardized prevalence rates (ASPRs) ranked similarly across countries in the Western Pacific region in 2021, with the highest in American Samoa and the lowest in Fiji. There were no surprises that incidence and prevalence increased in all countries. The EAPCs for incidence ranged from 0.24 (95% UI: 0.18–0.3) in Japan to 0.74 (95% UI: 0.58–0.91) in China. The EAPCs for prevalence ranged from 0.08 (95% UI: 0.05–0.1) in Cambodia to 0.91 (95% UI: 0.86–0.95) in Brunei Darussalam, with China ranking fifth at 0.67 (95% UI: 0.45–0.9) (Figure 1A). Since the large population, China has the highest total MASLD‐related deaths as well as DALYs of all countries, whereas age‐standardized rates are remarkable at 1.15 deaths (95% UI: 0.8–1.59) per 100,000 and 27.81 (95% UI: 19.34–38.78), and improving with EAPCs of −0.58 (95% UI: −0.72 to −0.45) and −0.97 (95% UI: −1.11 to −0.83) (Table 1). Mongolia has the highest age‐standardized deaths rate (ASDR) at 12.12 (95% UI: 7.42–19.09) per 10,000 and DALYs at 273.86 (95% UI: 167.16–431.9), which are worsening (Table 1 and Table S3). For an overview of changes, the EAPCs for death and DALYs ranged from −1.99 (95% UI: −2.22 to −1.77) in Japan to 1.86 (95% UI: 1.72–2) in Australia, and −2.43 (95% UI: −2.65 to −2.22) in Japan to 1.71 (95% UI: 1.57–1.86) in Australia (Figure 1A, Table 1, and Table S3).

### 3.2 Burden of Nonmalignant MASLD in the Western Pacific Region

Nonmalignant MASLD comprised almost all of the incidence and prevalent cases of total MASLD in the West Pacific region in 2021, with ASIR and ASPR being 814.16 (95% UI: 564.22–1113.66) and 20430.58 (95% UI: 15786.7–25741.27) per 100,000, respectively. Regional deaths due to nonmalignant MASLD in 2021 were estimated to account for less than half of the total at 10,195 (95% UI: 5475–17120) and were even lower after age standardization adjustment. The burden of death improved significantly, with an EAPC of −1.66 (95% UI: −1.79 to −1.52). The disparity between DALYs for nonmalignant and total MASLD was even apparent, 12.45 (95% UI: 6.57–21.12) for ASRs in 2021 and −1.84 (95% UI: −1.98 to −1.71) for EAPC from 1990 to 2021. (Table 1 and Tables S4–S9).

**FIGURE 2:**
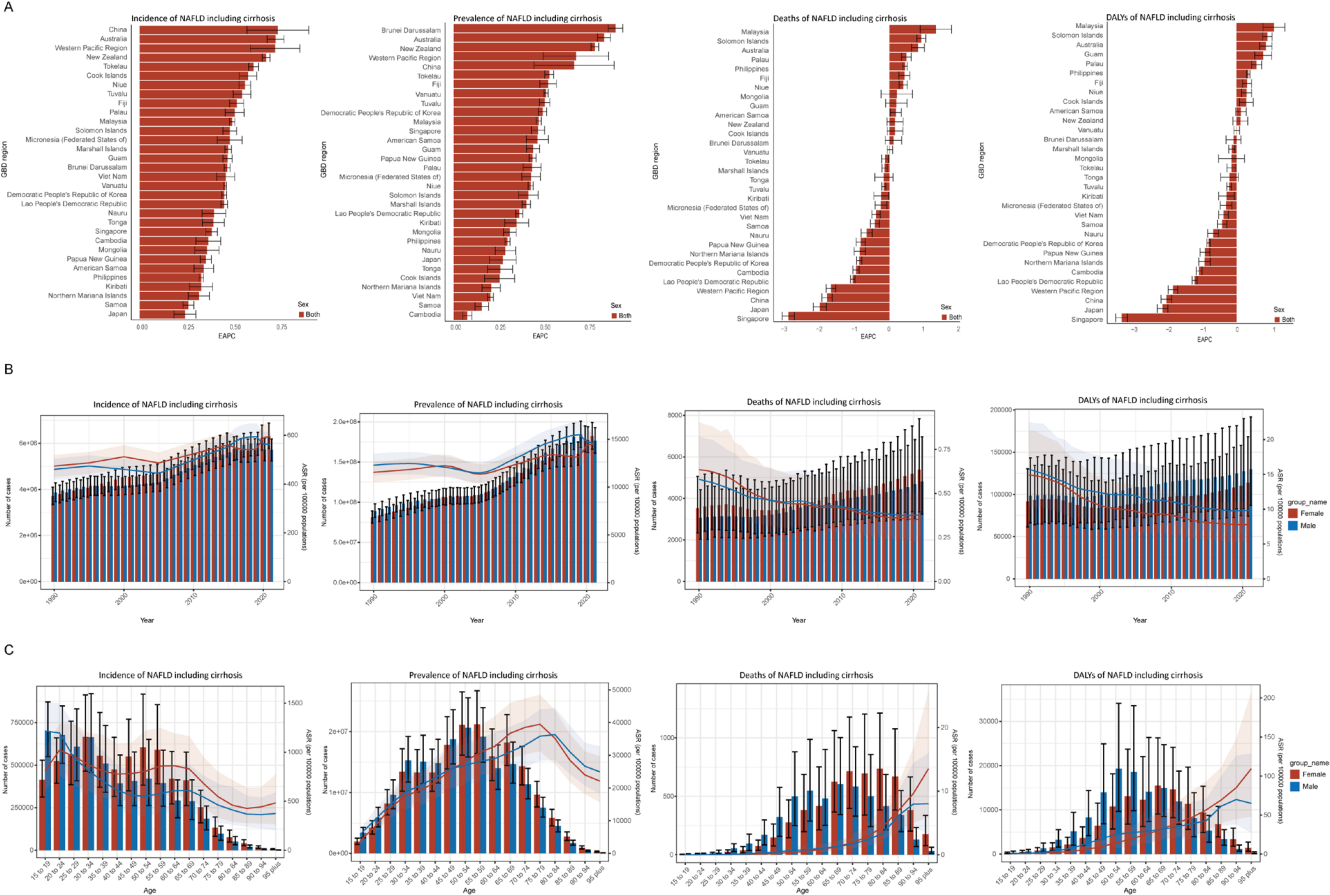
Characterization of nonmalignant MASLD burden in the Western Pacific region in 1990–2021. (A) Estimated annual percentage changes in age standardized rates of incidence, prevalence, deaths, and DALYs for nonmalignant MASLD by country, for the period 1990–2021. An EAPC of less than 0 represents a decrease, while greater than 0 indicates an increase, and an uncertainty interval spanning 0 is considered a nonsignificant result. (B) Changes in incidence, prevalence, deaths, and DALYs of nonmalignant MASLD by year in the Western Pacific between 1990 and 2021. Bar lengths represent values for the current year, and line positions indicate values after age‐standardization. (C) Changes in incidence, prevalence, deaths, and DALYs of nonmalignant MASLD by age in the Western Pacific between 1990 and 2021. Bar lengths represent values for the current year, and line positions indicate values after age‐standardization. DALY, disability adjusted life year; EAPC, estimated annual percentage change; GBD, global burden of disease.

As expected, the temporal and age distributions of incidence and prevalence of nonmalignant MASLD across genders were similar to the total (Figures 1 and 2). YLDs were slightly higher in women than men (0.28 vs. 0.22 in 2021), but the gap for other indicators seems to be narrowed (Figure 2B and Table S10). Transitions of male deaths and DALYs for nonmalignant MASLD after age 90 years flattened in comparison, and no shift was found in YLDs. ASR of YLDs in men with nonmalignant MASLD continued to rise, and the deaths, DALYs, and YLLs in men > 90 years turned out to be more moderate compared to the total burden (Figure 2C and Figure S2). In terms of deaths, the ASDR for males changed from 7.93 (95% UI: 4.35–14.36) cases per 100,000 in the 90–94‐year group to 8.03 (95% UI: 3.29–15.99) in the over 95‐year group, while for females it changed from 9.62 (95% UI: 5.05–16.71) to 13.52 (95% UI: 5.9–25.64) (Table S11).

The relative risks of incidence and prevalence of nonmalignant MASLD in 2021 and the 1990–2021 EAPCs for countries in the Western Pacific region are nearly identical to those for total MASLD (Figures 1 and 2, Tables S1, S2, S4, and S5). ASDRs in 2021 ranged between 0.18 (95% UI: 0.07–0.36) in the Cook Islands and 3.88 (95% UI: 1.9–6.93) in Mongolia, and the variation in Cook Island's rankings between total and nonmalignant MASLD (14/31 to 1/31) may be attributed to the low prevalence and long duration of disease (Table 1). Relative death risks in some countries do not stand out among the 31 countries in the Western Pacific but have progressed rapidly over the past 3 decades, particularly in Australia and Malaysia, with EAPCs of 0.81 (95% UI: 0.63–0.99) and 1.32 (95% UI: 0.87–1.76), respectively. By contrast, China, Japan, and Singapore appear well controlled, with EAPC values ranging from −1.77 to −2.89 (Figure 2A and Table 1). The distribution of the burden of DALYs and YLLs for nonmalignant MASLD across countries was essentially parallel to that of deaths (Figure 2A and Figure S2A), with ASR of DALYs ranging from 5.87 (95% UI: 2.98–10.3) to 93.36 (95% UI: 45.44–165.46) and ASR of YLLs ranging from 5.63 (95% UI: 2.85–9.87) to 92.27 (95% UI: 44.76–163.8). More than half of countries' DALYs improved compared to 1990, with Malaysia having the highest EAPC of 1.08 (0.76, 1.39). Seventeen out of 31 countries have improved their DALYs compared to 1990, whereas Malaysia failed and had the highest EAPC of 1.08 (95% UI: 0.76–1.39) (Tables S6 and S9).

### 3.3 Age‐Period‐Cohort Effects for Deaths of Total and Nonmalignant MASLD

**FIGURE 3:**
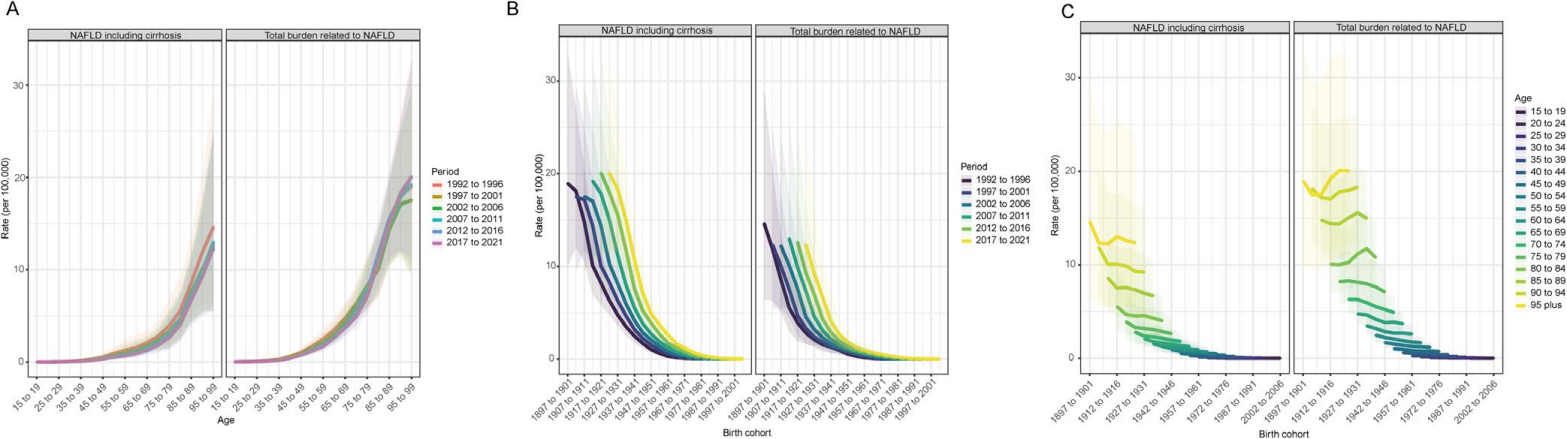
Age‐period‐cohort effects on deaths of nonmalignant and total MASLD in the Western Pacific, from 1990 to 2021. (A) Period deaths rates by age groups. (B) Period deaths rates by birth cohort. (C) Age groups deaths rates by birth cohort.

Our longitudinal age curves suggest that the burden of death from nonmalignant MASLD begins to increase significantly around age 35 years and slightly earlier for overall MASLD. The mortality is highest for those aged 95 years and older, with an overall MASLD mortality rate of 20.08 cases (95% UI: 11.04–32.06) per 100,000 people in 2017–2021 and 12.36 (95% UI: 5.53–24.01) for nonmalignant MASLD (Table 2). The rate of increase has a distinct fold in nonmalignant MASLD patients at age 80 years, after which the risk of death rises significantly, and a higher risk of death was observed in the 1992–1996 period (from 5.54 to 14.62 cases per 100,000, refer to Table 2). By contrast, the mortality of total MASLD slowed at the age of 90 and seemed to vary between periods, but the rate of slowing was not parallel to the period sequence (Figure 3A). For birth cohort effects, there was an overall downward trend in overall or nonmalignant MASLD among those aged 15–80. The risk of death fluctuates in those older than 80 years and is more dramatic in the overall MASLD burden (Figure 3C). Correlations between period and birth cohort were relatively clear and similar between nonmalignant and overall NAFLD; mortality increased with period progression, with the highest relative risk of death in the 2017–2021 period across birth cohort groups (Figure 3B). It is noteworthy that the ASIRs and ASPRs for total and nonmalignant MASLD are comparatively elevated in the 15–19 and 20–24 age cohorts (refer to Table S11). In contrast, the ASDRs and ASRs of DALYs within these groups are comparatively low. Overall, these indicators for males are higher than those for females among adolescents and young adults.

**TABLE 2:** Deaths rate per 100,000 population for total and nonmalignant MASLD corresponding to age and period.

Age (years)	Total MASLD						Nonmalignant MASLD					
	1992–1996	1997–2001	2002–2006	2007–2011	2012–2016	2017–2021	1992–1996	1997–2001	2002–2006	2007–2011	2012–2016	2017–2021
15–19	0.03 (0.02, 0.04)	0.03 (0.02, 0.04)	0.02 (0.02, 0.03)	0.02 (0.01, 0.03)	0.02 (0.01, 0.03)	0.02 (0.01, 0.03)	0.01 (0, 0.03)	0.01 (0, 0.02)	0.01 (0, 0.02)	0.01 (0, 0.01)	0.01 (0, 0.01)	0.01 (0, 0.01)
20–24	0.05 (0.03, 0.06)	0.04 (0.03, 0.06)	0.04 (0.03, 0.05)	0.03 (0.02, 0.04)	0.03 (0.02, 0.04)	0.03 (0.02, 0.04)	0.02 (0.01, 0.03)	0.02 (0.01, 0.03)	0.01 (0.01, 0.02)	0.01 (0.01, 0.02)	0.01 (0.01, 0.02)	0.01 (0.01, 0.02)
25–29	0.07 (0.05, 0.1)	0.07 (0.05, 0.1)	0.05 (0.04, 0.08)	0.05 (0.03, 0.07)	0.05 (0.04, 0.08)	0.06 (0.04, 0.08)	0.03 (0.01, 0.05)	0.02 (0.01, 0.04)	0.02 (0.01, 0.04)	0.02 (0.01, 0.04)	0.02 (0.01, 0.04)	0.02 (0.01, 0.05)
30–34	0.15 (0.11, 0.2)	0.15 (0.11, 0.19)	0.11 (0.08, 0.15)	0.1 (0.08, 0.14)	0.11 (0.08, 0.15)	0.11 (0.08, 0.16)	0.06 (0.03, 0.1)	0.05 (0.03, 0.09)	0.05 (0.03, 0.08)	0.05 (0.03, 0.08)	0.05 (0.03, 0.08)	0.05 (0.03, 0.08)
35–39	0.29 (0.2, 0.42)	0.28 (0.19, 0.39)	0.24 (0.16, 0.34)	0.21 (0.15, 0.3)	0.2 (0.13, 0.28)	0.21 (0.14, 0.3)	0.14 (0.06, 0.25)	0.12 (0.05, 0.22)	0.11 (0.05, 0.2)	0.1 (0.04, 0.17)	0.09 (0.04, 0.16)	0.1 (0.04, 0.17)
40–44	0.61 (0.43, 0.85)	0.56 (0.4, 0.76)	0.48 (0.34, 0.66)	0.44 (0.32, 0.61)	0.4 (0.28, 0.55)	0.41 (0.29, 0.57)	0.31 (0.17, 0.51)	0.26 (0.14, 0.43)	0.23 (0.12, 0.39)	0.21 (0.11, 0.35)	0.19 (0.1, 0.31)	0.19 (0.1, 0.32)
45–49	1.03 (0.71, 1.49)	0.97 (0.69, 1.36)	0.83 (0.59, 1.18)	0.78 (0.55, 1.12)	0.79 (0.56, 1.11)	0.74 (0.52, 1.06)	0.54 (0.27, 0.98)	0.44 (0.22, 0.79)	0.41 (0.21, 0.74)	0.39 (0.2, 0.71)	0.36 (0.18, 0.65)	0.33 (0.17, 0.59)
50–54	1.69 (1.15, 2.4)	1.54 (1.08, 2.15)	1.41 (1, 1.93)	1.29 (0.91, 1.79)	1.25 (0.88, 1.71)	1.2 (0.83, 1.67)	0.95 (0.49, 1.63)	0.76 (0.39, 1.29)	0.68 (0.35, 1.14)	0.62 (0.32, 1.07)	0.54 (0.28, 0.92)	0.51 (0.26, 0.87)
55–59	2.48 (1.75, 3.57)	2.24 (1.6, 3.15)	2.07 (1.51, 2.83)	1.89 (1.36, 2.58)	1.76 (1.27, 2.4)	1.68 (1.19, 2.37)	1.28 (0.67, 2.29)	1.07 (0.55, 1.91)	0.93 (0.48, 1.62)	0.83 (0.43, 1.48)	0.75 (0.39, 1.31)	0.69 (0.37, 1.23)
60–64	3.48 (2.56, 4.88)	3.2 (2.36, 4.36)	2.86 (2.13, 3.91)	2.71 (2, 3.71)	2.7 (1.98, 3.69)	2.61 (1.89, 3.63)	1.56 (0.81, 2.95)	1.29 (0.68, 2.41)	1.18 (0.62, 2.21)	1.11 (0.59, 2.07)	0.99 (0.51, 1.8)	0.93 (0.48, 1.68)
65–69	4.76 (3.62, 6.32)	4.66 (3.59, 6.08)	4.19 (3.25, 5.44)	3.84 (2.93, 5.01)	3.89 (2.96, 5.06)	3.71 (2.74, 4.91)	2.1 (1.31, 3.35)	1.79 (1.13, 2.81)	1.65 (1.03, 2.58)	1.53 (0.95, 2.37)	1.37 (0.85, 2.12)	1.28 (0.77, 2.02)
70–74	6.32 (4.78, 8.46)	6.3 (4.85, 8.18)	5.96 (4.58, 7.75)	5.54 (4.19, 7.25)	5.23 (3.98, 6.93)	4.91 (3.62, 6.5)	2.8 (1.63, 4.52)	2.4 (1.4, 3.8)	2.31 (1.34, 3.62)	2.16 (1.26, 3.4)	1.96 (1.15, 3.12)	1.81 (1.05, 2.88)
75–79	8.21 (5.81, 11.57)	8.29 (6.08, 11.4)	8.15 (5.99, 10.96)	7.99 (5.87, 10.84)	7.64 (5.62, 10.37)	7.11 (5.13, 9.65)	3.91 (1.93, 6.78)	3.31 (1.64, 5.65)	3.19 (1.58, 5.44)	3.09 (1.55, 5.27)	2.86 (1.44, 4.9)	2.64 (1.33, 4.45)
80–84	10.08 (6.97, 14.44)	10.02 (7.09, 13.97)	10.27 (7.28, 13.89)	11.19 (8.05, 15.03)	11.73 (8.44, 15.75)	10.79 (7.73, 14.52)	5.54 (2.96, 9.16)	4.7 (2.57, 7.79)	4.54 (2.52, 7.39)	4.57 (2.52, 7.43)	4.3 (2.34, 7.02)	4.03 (2.17, 6.55)
85–89	14.8 (10.54, 20.71)	14.4 (10.57, 19.45)	14.38 (10.52, 19.21)	15.04 (11.04, 20.04)	15.57 (11.38, 20.52)	14.99 (10.8, 19.79)	8.6 (5.04, 13.48)	7.52 (4.44, 11.73)	7.58 (4.47, 11.83)	7.34 (4.33, 11.56)	6.96 (4.1, 10.99)	6.69 (3.93, 10.66)
90–94	18.13 (12.03, 26.92)	17.2 (11.69, 24.96)	17.04 (11.48, 24.91)	17.84 (11.98, 25.73)	17.98 (12.03, 25.62)	18.32 (12.15, 26.36)	11.87 (6.3, 20.5)	10.06 (5.41, 17.59)	10.08 (5.52, 17.64)	9.88 (5.38, 17.3)	9.3 (5.12, 16.27)	9.22 (5.05, 16.21)
95 plus	18.96 (9.97, 33.15)	17.49 (9.48, 29.37)	17.55 (9.47, 29.45)	19.22 (10.42, 31.96)	20.09 (11.13, 32.56)	20.08 (11.04, 32.06)	14.62 (6.39, 29.14)	12.35 (5.54, 24.9)	12.25 (5.52, 24.15)	13.01 (5.97, 25.55)	12.64 (5.82, 24.8)	12.36 (5.53, 24.01)

Abbreviation: MASLD, metabolic dysfunction‐associated steatotic liver disease.

### 3.4 Predicted Total and Nonmalignant MASLD Burden in Specific Countries

**FIGURE 4:**
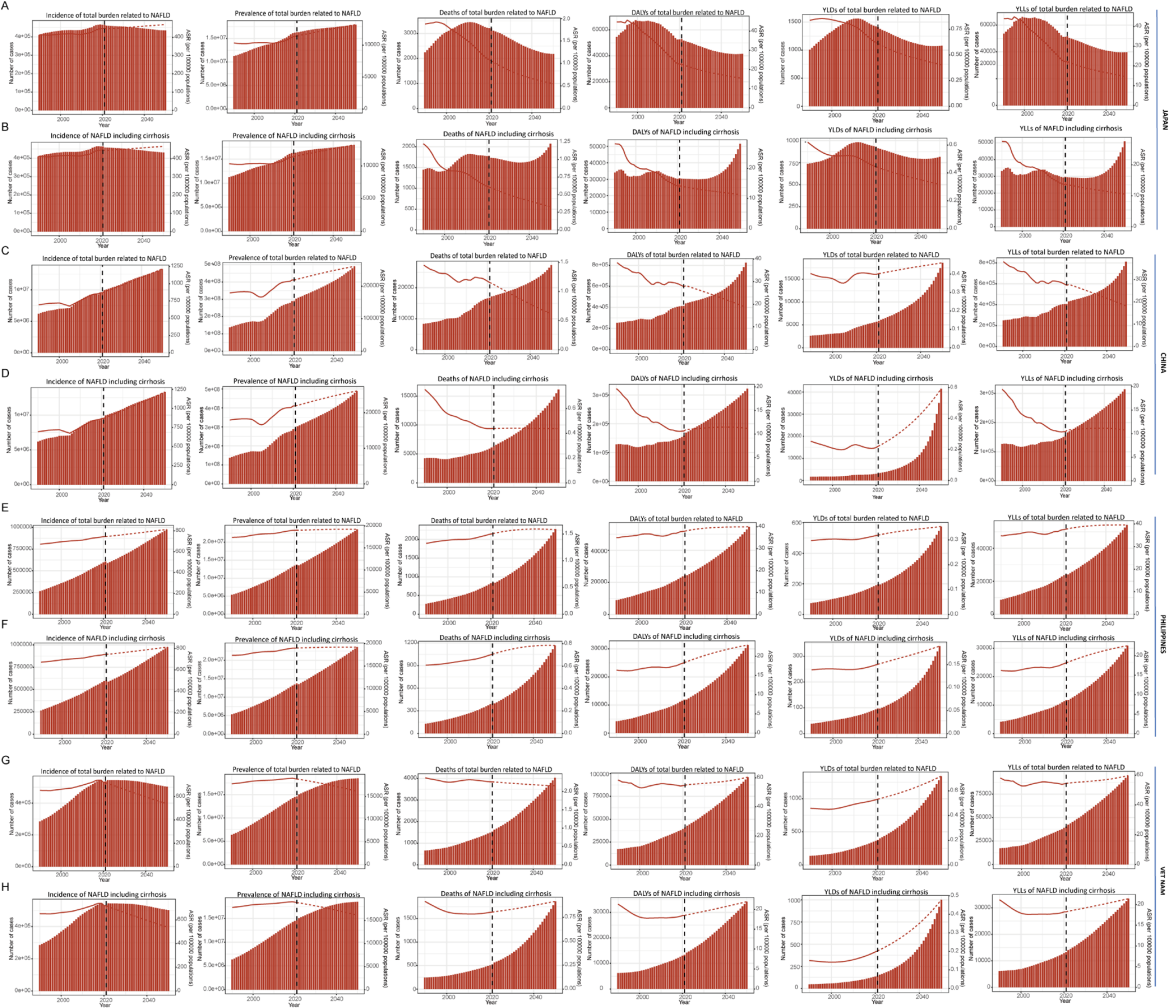
Bayesian Age‐period‐cohort forecasting models for nonmalignant and total MASLD in Japan (A‐B), China (C‐D), the Philippines (E‐F), and Viet Nam (G‐H). The BAPC model was trained for the period 1990–2021 and projected for the period 2022–2050. The first row for each country is the total MASLD burden, which includes, in order, incidence, prevalence, deaths, DALYs, YLDs, and YLLs, while the second row is the nonmalignant MASLD burden. Bar lengths represent deaths value for the current year, and line positions indicate age standardized deaths rate value.

Our study also predicts the burden trends of total and nonmalignant MASLD, which ends in 2050 (Figure 4 and Table S12). Over the next 30 years, Viet Nam will be the only country with a sustained decrease in the ASIR and ASPR of total MASLD. The increases in Japan and the Philippines will be slower, while there will be a notable rise in China. ASIRs in 2050 are estimated to be 719.58 (95% UI: 717.67–721.49), 470.21 (95% UI: 144.83–795.6), 813.77 (95% UI: 353.47–1274.06), and 1213.46 (95% UI: 128.22–2298.71), respectively. There is no significant improvement in the ASRs of deaths or DALYs in Viet Nam. On the contrary, China has a significant reduction, with ASDR estimated to be 0.62 (95% UI: −0.46 to 1.69) and the ASR of DALYs to be 18.73 (95% UI: −21.1 to 58.56) in 2050. Japan and the Philippines are as expected, with declines and mild increases, respectively.

The incidence and prevalence of nonmalignant MASLDs are parallel and differ little from the total MASLD picture from 2021 to 2050. The ASRs of deaths and DALYs continue to decline in Japan, with the ASDR changing from 0.61 (95% UI: 0.58–0.63) in 2021 to 0.33 (95% UI: −0.34 to 0.99) in 2050, and the ASR of DALYs decreasing from 14.12 (95% UI: 13.95–14.29) to 11.26 (95% UI: −15.27 to 37.79). The absolute risk of death and DALYs declines in Japan, but turns around 2035 and rises rapidly. Unlike the other three countries, projected trends of YLDs in Japan had significant differences between nonmalignant and total MASLD, while the differences in YLLs were modest. Chinese relative risks of deaths, DALYs, and YLLs remain stable over the next 30 years, in which ASRs of deaths and DALYs in 2050 are 0.44 (95% UI: −0.2 to 1.07) and 11.22 (95% UI: −3.91 to 26.35). The increase in YLDs in China is very striking, with absolute and relative risks in 2050 compared to 2021 increasing by approximately 13‐folds and 2.6‐folds, respectively. The rough trends in the burden of nonmalignant MASLD in the Philippines and Vietnam are comparable, with relative risk in the Philippines slowing increase and in Viet Nam evenly.

## 4 Discussion

Several recently published meta‐analyses have included only selected countries in the Western Pacific region, reporting an incidence of 47.3–60.85 per 1000 persons‐year in China, 24.37–39.5 in Japan, and 41.59–60.2 in South Korea, while general knowledge of the burden of MASLD in the Western Pacific is lacking (Riazi et al. 2022; Le et al. 2023). The characterization of disease burden in countries with limited academic activity often depends on GBD studies, especially in certain island nations. This study presents an updated analysis of the disease burden associated with MASLD, including its nonmalignant aspects, within the Western Pacific region.

The absolute burden of MASLD in the Western Pacific region is both substantial and increasing. In 2021, the total prevalence of MASLD was estimated at 359.2 million cases, with mortality reaching 25,300 cases. Notably, nonmalignant MASLD accounted for nearly the entire prevalence and approximately 40% of the mortality rate. MASLD is classified as a metabolic syndrome. In addition to liver‐related metabolic disturbances, it frequently coexists with metabolic abnormalities in other systems, including the cardiovascular system and endocrine system, which significantly contribute to morbidity and mortality among patients with non‐cirrhotic MASLD (Konyn et al. 2022). It should be noted that MASLD is associated with a significantly elevated risk of mortality following the onset of disability, particularly during the cirrhotic decompensation stage, while YLLs in nonmalignant MASLD are generally small. Furthermore, disability weights for nonmalignant MASLD are low, with a 0.32 rating for decompensation with severe heart failure, slightly higher than the 0.29 at initial NASH‐associated HCC diagnosis, and much lower than advanced type 2 diabetes. Therefore, early management of MASLD patients in the nonmalignant phase is vital for disease prognosis. Clinicians can utilize advanced imaging and serological techniques to identify early hepatic lesions and metabolic disorders in patients. Besides, physicians can also assist patients in reversing the progression of the disease by promoting their lifestyle modifications through educational interventions and pharmacological treatments. Policymakers can raise public awareness of this disease through media coverage. It is imperative for public health institutions to systematically monitor disease progression across various countries and regions in a timely fashion, while ensuring prompt communication with policymakers and medical institutions.

According to the prediction results of BAPC, the incidence and prevalence of nonmalignant or overall MASLD will continue to increase in selected countries. The prevalence of nonmalignant MASLD in China is projected to increase by approximately 1.7 times in 2050 compared to 2021. Japan stands out as the sole country where the absolute risk of morbidity is expected to decline. The ASIR in Japan is also anticipated to rise at the slowest pace. The results of this prediction piqued our interest and prompted further contemplation. On one hand, Japan's healthcare system is recognized as one of the most advanced in the Western Pacific region. On the other hand, while Japanese culinary traditions bear similarities to those of China, factors such as geographical location, government policies, and limitations in ingredient diversity contribute to a dietary pattern in Japan that is characterized by lighter and healthier choices, including lower calorie intake, as well as higher protein consumption (Klenk et al. 2016). Another explanation for this trend could be the demographic contraction observed within Japanese society (Estes et al. 2018). Another important finding is that our projections indicate an increasing contribution of death and disability associated with nonmalignant MASLD (Tacke et al. 2024). A milestone drug for MASLD is a thyroid hormone receptor β agonist (Resmetirom) (Harrison et al. 2024, 2019), which is the world's first drug approved by the US Food and Drug Administration (FDA) for the treatment of MASH. Studies have shown that this drug can promote the metabolism of fatty acids in the liver and reduce inflammatory responses and fibrosis in the liver to a certain extent. However, targeted therapeutic options for MASLD remain limited, prompting ongoing efforts by medical professionals to develop multistage and efficacious pharmacological interventions.

Another important finding of our study is the elevated incidence of MASLD among younger age cohorts. Notably, the Western Pacific region exhibits a significant prevalence of MASLD among adolescents and young adults, particularly males aged 15–24. While the ASDRs and ASRs of DALYs in these cohorts are comparatively low, the influence of MASLD on life expectancy and health outcomes is expected to progressively intensify with advancing age. Fortunately, the ASDRs and ASRs of DALYs associated with nonmalignant MASLD are relatively low, which provides adolescents with the opportunity for prompt intervention and treatment. The rising rates of childhood obesity are a major factor contributing to the increased prevalence of MASLD in young populations in Western countries and have recently garnered attention in the Western Pacific region (Wong et al. 2023). Although MASLD is not a significant cause of death from chronic liver disease in younger populations (Paik et al. 2022), the impact of MASLD on death and disability is not likely to be apparent until much later. Future objectives of governments and clinicians are anticipated to include the management of obesity and the promotion of healthy living awareness among children and adolescents. For example, body mass index screening in children and adolescents is an important tool to combat obesity worldwide. Specifically, national strategies may include intensive behavioral therapy to change diet and exercise; screening for and management of hypertension, glucose intolerance, dyslipidaemia, and abnormal liver function in children and adolescents with obesity; and in extreme cases bariatric surgery.

Intuitively, highly developed countries with well‐managed noncommunicable diseases (NCDs) are supposed to perform better in reducing the burden of MASLD and vice versa. We thus make an interesting comparison of our results with the MASLD‐Sustainable Development Goal index (developed by Lazarus et al. for characterizing national preparedness to deal with the MASLD challenge) and NCD burdens (Man et al. 2023; The Global Health Observatory, n.d.). Australian ASDR for NCD was 278.5 cases per 100,000 in 2019, and it scored high in MASLD‐SDG, indicating favorable practices of NCD management and that they are well developed. However, our analyses revealed a very high and rapidly deteriorating burden of death from MASLD in Australia. Although the significant variation in some countries (e.g., Papua New Guinea) might be ascribed to the limited data available, we believe this inconsistency could indicate the particularities in the management of MASLD. Another potential factor contributing to the progression of MASLD in Australia is dietary variation. The Western dietary pattern is prevalent in Australia and is characterized by a high intake of fats. Western Pacific countries do not appear to be ready for growing MASLD. Unlike cardiovascular disease, diabetes, and even obesity, as of 2020, countries in the Western Pacific region (which includes 12 major countries) have no national strategies for MASLD and have published limited specific clinical guidelines (Lazarus et al. 2022). In Japan, a national program called Specific Health Check‐ups was initiated in fiscal year 2008 to prevent metabolic disorders and lifestyle‐induced diseases; however, there appears to be no significant improvement in the prevalence of MASLD (Sato et al. 2024). China released a “Healthy China (HC 2030) strategy” in 2016 that emphasizes disease prevention, healthcare system reform, and policy building (Tan et al. 2017). In 2025, the National Health Commission of China proposed the implementation of a 3‐year initiative entitled the “Weight Management Year” which aims to promote healthy lifestyle practices and enhance the prevention and management of chronic diseases. Specific measures involve encouraging individuals to pursue scientifically informed weight loss strategies and facilitating the advancement of health innovation industries. However, MASLD diagnostic criteria may limit the transfer of MASLD management to primary care, and related measures may be complex to advance because of patients' and healthcare providers' complaints (Nan et al. 2021). The impact of the Healthy China strategy on MASLD improvement is expected to require longer time scales to assess. Public awareness is another aspect. Awareness of MASLD is generally lacking and varies widely between countries in the Western Pacific region (Peng et al. 2024). For instance, in high‐income nations like South Korea, the general populace possesses a substantial comprehension of the fundamental aspects of MASLD. However, there is a deficient awareness regarding the extrahepatic complications associated with MASLD, and the propensity to pursue medical intervention remains low. Conversely, in low‐income countries, there exists a paucity of basic knowledge concerning MASLD, attributable to limited access to medical education and public scientific outreach (Lee et al. 2023).

While our study contributes to addressing the knowledge gaps concerning the current burden and future trends of MASLD in the Western Pacific, we acknowledge the presence of certain limitations. Firstly, the interpretation of the disease is influenced by the GBD data framework. On the one hand, it limits further analyses of the burden of varied MASLD stages and cardiac risk factor subgroups. On the other hand, the burden of death in nonmalignant MASLD may be underestimated, as mentioned earlier. Secondly, GBD studies are affected by the accessibility of primary data. In countries with less robust data sources, the GBD outcomes were predominantly characterized by imputation, potentially introducing substantial bias into our secondary analyses. This complicates the interpretation of MASLD outcomes in smaller nations within the Western Pacific region. Besides, the improvement of MASLD deaths and DALYs in our analysis may be attributed to the estimation methodology of the GBD study, and worsening trends in MASLD may be masked by improvements in HBV or HCV (Paik et al. 2023), and thus need to be interpreted with caution. Last but not least, the GBD 2021 study was influenced by the Covid‐19 pandemic, resulting in delays and gaps in data acquisition, as well as an increase in mortality attributable to Covid‐19, which further complicates the interpretation of mortality statistics (Schumacher et al. 2024).

In conclusion, our study highlights that the burden of MASLD in the Western Pacific is severe and continues to rise. MASLD is becoming more common in younger people, potentially increasing future disease burden. Moreover, nonmalignant MASLD will increasingly become the main focus of MASLD prevention and control, posing significant challenges for policy and healthcare. Raising awareness among stakeholders and the public early is crucial to address these upcoming risks.

## Author Contributions

**Qichao Ge:** conceptualization (lead), data curation (lead), formal analysis (lead), methodology (lead), project administration (equal), writing – original draft (lead). **Yuan Lin:** data curation (equal), methodology (equal), writing – original draft (equal), writing – review and editing (equal). **Mingwang Wang:** data curation (equal), formal analysis (equal), methodology (equal). **Jianwei Zhu:** data curation (supporting). **Qingqing Zhang:** data curation (supporting). **Junjun Wang:** investigation (supporting), writing – review and editing (equal). **Yufei Yang:** validation (supporting). **Hanjing Zhangdi:** supervision (equal), validation (equal), writing – review and editing (equal). **Yuecheng Guo:** investigation (lead), supervision (equal), validation (equal). **Shanjuan Wang:** funding acquisition (equal), project administration (equal), supervision (equal). **Lungen Lu:** funding acquisition (lead), project administration (lead), supervision (lead), validation (lead).

## Ethics Statement

This study uses de‐labeled data from GBD 2021, which is open access and available for noncommercial purposes, subject to the data use terms as stated by IHME (http://ghdx.healthdata.org).

## Conflicts of Interest

The authors declare no conflicts of interest.

## Supporting information


Appendix S1.

## Data Availability

The data from this study can be accessed openly through the GBD 2021 online database, as outlined in the Section 2.
