# Supplementary material for: The Burden of Nonmalignant Metabolic Dysfunction‐Associated Steatotic Liver Disease in the Western Pacific Region: A Systematic Analysis From 1990 to 2021
Source: Food Sci Nutr. 2025 Jul 16;13(7):e70627. doi: 10.1002/fsn3.70627 (PMC12267884; doi:10.1002/fsn3.70627)

**The Burden of Non-malignant Metabolic Dysfunction-Associated**

**Steatotic Liver Disease In the Western Pacific Region:**

**A Systematic Analysis From 1990 to 2021**

**Supplementary appendix 1**

[**Table S1.** Incident cases and age-standardized incidence rate (ASIR) for total MASLD in 31 West Pacific countries in 1990 and 2021, and estimated annual percentage changes (EAPC) of ASIR cross 1990-2021. 2](#TableS1)

[**Table S2.** Prevalent cases and age-standardized prevalence rate (ASPR) for total MASLD in 31 West Pacific countries in 1990 and 2021, and estimated annual percentage changes (EAPC) of ASPR cross 1990-2021. 3](#TableS3)

[**Table S3.** Disability-adjusted life years (DALYs) and age-standardized rate of DALYs (ASR-DALYs) for total MASLD in 31 West Pacific countries in 1990 and 2021, and estimated annual percentage changes (EAPC) of ASR-DALYs cross 1990-2021. 4](#TableS6)

[**Table S4.** Incident cases and age-standardized incidence rate (ASIR) for non-malignant MASLD in 31 West Pacific countries in 1990 and 2021, and estimated annual percentage changes (EAPC) of ASIR cross 1990-2021. 5](#TableS2)

[**Table S5.** Prevalent cases and age-standardized prevalence rate (ASPR) for non-malignant MASLD in 31 West Pacific countries in 1990 and 2021, and estimated annual percentage changes (EAPC) of ASPR cross 1990-2021. 6](#TableS4)

[**Table S6.** Disability-adjusted life years (DALYs) and age-standardized rate of DALYs (ASR-DALYs) for non-malignant MASLD in 31 West Pacific countries in 1990 and 2021, and estimated annual percentage changes (EAPC) of ASR-DALYs cross 1990-2021. 8](#TableS7)

[**Table S7.** Deaths for total and non-malignant MASLD in 31 West Pacific countries in 1990 and 2021. 9](#TableS5)

[**Table S8.** Years lived with disability (YLDs) and age-standardized rate of YLDs (ASR-YLDs) for total and non-malignant MASLD in 31 West Pacific countries in 1990 and 2021, and estimated annual percentage changes (EAPC) of ASR-YLDs cross 1990-2021.10](#TableS8)

[**Table S9.** Years of life lost (YLLs) and age-standardized rate of YLLs (ASR-YLLs) for total and non-malignant MASLD in 31 West Pacific countries in 1990 and 2021, and estimated annual percentage changes (EAPC) of ASR-YLLs cross 1990-2021. 11](#TableS9)

[**Table S10.** Regional burdens of total and non-malignant MASLD by genders in 2021 14](#TableS11)

[**Table S11.** Regional burdens of total and non-malignant MASLD by genders and ages in 2021 14](#TableS12)

[**Table S12.** Predicted Burdens of total and non-malignant MASLD based on Bayesian age-period-cohort model in Japan, China, Philippines and Viet Nam in 2021 and 2050. 16](#TableS10)

[**Figure S1.** Years lived with disability (YLDs) and Years of life lost (YLLs) of total MASLD burden, by countries, years and ages, respectively, in the Western Pacific region in 1990–2021 19](#figureS1)

**[Figure S2.](#figureS2)** [Years lived with disability (YLDs) and Years of life lost (YLLs) of total MASLD burden, by countries, years and ages, respectively, in the Western Pacific region in 1990–2021 20](#figureS2)

**Table S1. Incident cases and age-standardized incidence rate (ASIR) for total MASLD in 31 West Pacific countries in 1990 and 2021, and estimated annual percentage changes (EAPC) of ASIR cross 1990-2021.**

| **Location** | **1990 incident cases(95%UI)** | **2021 incident cases(95%UI)** | **1990 ASIR per 100,000(95% UI)** | **2021 ASIR per 100,000(95% UI)** | **EAPC of ASIR (95% UI)** |
| --- | --- | --- | --- | --- | --- |
| American Samoa | 344 (239, 472) | 412 (281, 574) | 1036.07 (705.27, 1448.01) | 1156.89 (788.25, 1610.23) | 0.34 (0.29, 0.4) |
| Australia | 51542 (35339, 71058) | 96982 (65761, 135815) | 398.38 (274.09, 547.98) | 498.32 (344.98, 685.79) | 0.73 (0.69, 0.78) |
| Brunei Darussalam | 1062 (743, 1438) | 2410 (1636, 3330) | 596.74 (410.19, 820.09) | 686.2 (467.02, 946.14) | 0.47 (0.45, 0.48) |
| Cambodia | 38016 (26483, 51721) | 86569 (59962, 118296) | 648.14 (442.92, 896.56) | 711.68 (489.42, 977.35) | 0.37 (0.3, 0.43) |
| China | 6183807 (4321929, 8406882) | 9572435 (6471989, 13346890) | 695.08 (478.62, 955.89) | 871.91 (602.58, 1193.65) | 0.74 (0.58, 0.91) |
| Cook Islands | 106 (73, 145) | 123 (83, 173) | 801.49 (545.58, 1113.48) | 936.9 (638.38, 1302.66) | 0.58 (0.53, 0.63) |
| Democratic People's Republic of Korea | 99424 (68629, 136576) | 162783 (111239, 225255) | 668.17 (458.83, 921.81) | 767.23 (529.65, 1051.87) | 0.45 (0.44, 0.47) |
| Fiji | 4894 (3436, 6649) | 7560 (5239, 10405) | 956.54 (660.74, 1316.67) | 1138.63 (788.14, 1567.32) | 0.52 (0.48, 0.56) |
| Guam | 816 (567, 1114) | 1082 (742, 1499) | 803.4 (551.22, 1108.66) | 919.13 (635.3, 1264.38) | 0.47 (0.45, 0.5) |
| Japan | 410741 (284089, 566411) | 465784 (313199, 656187) | 413.88 (289.4, 565.46) | 449.44 (314.16, 613.75) | 0.24 (0.18, 0.3) |
| Kiribati | 447 (316, 603) | 880 (617, 1195) | 930.95 (645.99, 1275.13) | 1053.89 (732.25, 1442.64) | 0.33 (0.27, 0.39) |
| Lao People's Democratic Republic | 13953 (9687, 19045) | 34677 (23804, 47401) | 577.32 (394.06, 799.68) | 663.28 (450.77, 915.1) | 0.45 (0.43, 0.47) |
| Malaysia | 109963 (76790, 149420) | 267268 (183291, 369456) | 929.5 (638.89, 1281.19) | 1080.16 (739.98, 1494.67) | 0.5 (0.48, 0.51) |
| Marshall Islands | 194 (137, 262) | 365 (254, 501) | 768.2 (528.98, 1058.39) | 897.52 (619.31, 1242.33) | 0.47 (0.46, 0.49) |
| Micronesia (Federated States of) | 491 (344, 667) | 707 (491, 969) | 792.28 (542.7, 1097.47) | 932.87 (641.06, 1287.97) | 0.48 (0.42, 0.55) |
| Mongolia | 9506 (6760, 12757) | 17133 (11838, 23357) | 698.34 (484.96, 956.25) | 770.52 (533.68, 1046.47) | 0.38 (0.31, 0.44) |
| Nauru | 50 (35, 68) | 68 (48, 92) | 783.51 (534.39, 1084.07) | 900.61 (622.34, 1237.65) | 0.4 (0.33, 0.46) |
| New Zealand | 11314 (7841, 15537) | 20976 (14281, 29007) | 432.32 (299.92, 593.78) | 528.19 (365.52, 721.29) | 0.68 (0.66, 0.7) |
| Niue | 12 (9, 17) | 12 (8, 17) | 810.23 (559.38, 1129.38) | 970.82 (665.22, 1347.54) | 0.57 (0.53, 0.6) |
| Northern Mariana Islands | 328 (225, 452) | 365 (248, 507) | 916.2 (623.58, 1274.26) | 1018.76 (699.63, 1405.11) | 0.32 (0.26, 0.37) |
| Palau | 95 (66, 129) | 137 (93, 193) | 841.81 (578.04, 1161.82) | 991.63 (679.69, 1366.95) | 0.51 (0.46, 0.56) |
| Papua New Guinea | 17870 (12633, 24141) | 53452 (37686, 72106) | 702.7 (486.86, 966) | 777.67 (541.16, 1060.09) | 0.35 (0.32, 0.38) |
| Philippines | 266101 (186923, 359546) | 606856 (421068, 828938) | 672.36 (461.63, 926.36) | 745.58 (512.75, 1026.07) | 0.33 (0.32, 0.34) |
| Samoa | 904 (638, 1229) | 1287 (901, 1767) | 827.19 (568.05, 1151.2) | 909.21 (628.86, 1260.65) | 0.26 (0.23, 0.29) |
| Singapore | 14652 (10181, 20066) | 29991 (19986, 42588) | 597.13 (412.17, 822.52) | 682.42 (470.18, 943.24) | 0.39 (0.35, 0.42) |
| Solomon Islands | 1454 (1026, 1951) | 3834 (2672, 5198) | 738.71 (508.81, 1012.63) | 855.21 (586.81, 1172.96) | 0.48 (0.45, 0.52) |
| Tokelau | 8 (6, 11) | 9 (6, 12) | 773.09 (531.39, 1057.51) | 925.51 (642.15, 1271.31) | 0.61 (0.58, 0.64) |
| Tonga | 539 (382, 726) | 703 (488, 960) | 857.99 (593.35, 1178.65) | 990.69 (679.11, 1364.18) | 0.39 (0.33, 0.45) |
| Tuvalu | 47 (33, 64) | 81 (56, 111) | 752.43 (519.87, 1034.59) | 909.46 (625.18, 1248.83) | 0.55 (0.5, 0.6) |
| Vanuatu | 800 (563, 1077) | 2133 (1501, 2896) | 894.19 (617.21, 1223.49) | 1027.66 (714.26, 1408.88) | 0.46 (0.45, 0.47) |
| Viet Nam | 285189 (200136, 384627) | 536533 (367637, 740806) | 652.37 (449.61, 893.29) | 719.81 (496.71, 987.28) | 0.46 (0.41, 0.51) |
| Western Pacific Region | 7588600 (5309241, 10303631) | 12039002 (8184084, 16729865) | 656.24 (453.26, 900.13) | 814.99 (565.02, 1114.5) | 0.73 (0.59, 0.86) |

**Table S2. Prevalent cases and age-standardized prevalence rate (ASPR) for total MASLD in 31 West Pacific countries in 1990 and 2021, and estimated annual percentage changes (EAPC) of ASPR cross 1990-2021.**

| **Location** | **1990 prevalent cases(95%UI)** | **2021 prevalent cases(95%UI)** | **1990 ASPR per 100,000(95% UI)** | **2021 ASPR per 100,000(95% UI)** | **EAPC of ASPR (95% UI)** |
| --- | --- | --- | --- | --- | --- |
| American Samoa | 6059 (4656, 7655) | 10312 (8043, 12741) | 24896.66 (19271.52, 31220.15) | 28874.48 (22520.67, 35703.97) | 0.47 (0.41, 0.53) |
| Australia | 1372057 (1042200, 1760073) | 3141588 (2408560, 3961965) | 10272.24 (7799.76, 13178.11) | 13189.43 (10073.34, 16678.44) | 0.84 (0.81, 0.88) |
| Brunei Darussalam | 19626 (14887, 25140) | 59710 (45595, 75932) | 13554.51 (10294.38, 17363.26) | 17251.98 (13185.75, 21912.5) | 0.91 (0.86, 0.95) |
| Cambodia | 821554 (625720, 1044376) | 1959554 (1499862, 2497494) | 17798.63 (13582.14, 22614.08) | 17863.95 (13680.78, 22737.4) | 0.08 (0.05, 0.1) |
| China | 137118102 (105146942, 174341115) | 291261235 (225509637, 366315528) | 17933.73 (13769.59, 22770.07) | 21887.22 (16888.11, 27588.61) | 0.67 (0.45, 0.9) |
| Cook Islands | 2492 (1916, 3148) | 3764 (2929, 4694) | 22809.94 (17591.73, 28723.58) | 24277.87 (18816.71, 30421.46) | 0.26 (0.17, 0.34) |
| Democratic People's Republic of Korea | 2338362 (1784779, 2988102) | 4522087 (3482184, 5734985) | 16992.1 (12982.99, 21715.48) | 19655.02 (15134.36, 24937.36) | 0.5 (0.47, 0.52) |
| Fiji | 96312 (73778, 122317) | 179996 (139160, 225682) | 23868.72 (18329.32, 30182.58) | 28305.39 (21913.6, 35437.11) | 0.53 (0.48, 0.58) |
| Guam | 17123 (13116, 21817) | 31854 (24813, 39603) | 21204.93 (16319.34, 26857.1) | 23987.3 (18641.42, 29903.24) | 0.44 (0.41, 0.48) |
| Japan | 11269643 (8621266, 14372380) | 16312365 (12602387, 20682815) | 10325.08 (7893.4, 13175.98) | 11406.38 (8781.9, 14466.65) | 0.27 (0.2, 0.35) |
| Kiribati | 8438 (6458, 10754) | 18105 (13906, 22945) | 22462.09 (17231.99, 28510.64) | 25691.31 (19798.44, 32442.92) | 0.35 (0.29, 0.42) |
| Lao People's Democratic Republic | 294409 (224024, 378509) | 745099 (565761, 954998) | 15092.37 (11491.31, 19391.36) | 16615.68 (12648.32, 21255.72) | 0.37 (0.35, 0.39) |
| Malaysia | 2281044 (1737454, 2905218) | 6440219 (4971059, 8107446) | 23608.78 (18055.29, 29987.67) | 27365.91 (21161.6, 34412.97) | 0.48 (0.46, 0.49) |
| Marshall Islands | 3548 (2700, 4551) | 7610 (5821, 9674) | 19505.97 (14891.58, 24928.66) | 22070.52 (16951.09, 27946.4) | 0.41 (0.38, 0.43) |
| Micronesia (Federated States of) | 9394 (7174, 12011) | 15074 (11567, 19107) | 20053.06 (15358.54, 25568.54) | 23135.53 (17834.83, 29226.2) | 0.43 (0.38, 0.48) |
| Mongolia | 189464 (143997, 243491) | 417594 (318967, 532228) | 18157.88 (13842.14, 23270.25) | 19305.04 (14772.31, 24544.46) | 0.32 (0.28, 0.35) |
| Nauru | 983 (751, 1252) | 1287 (985, 1637) | 19847.24 (15207.24, 25210.3) | 21773.86 (16726.66, 27581.89) | 0.29 (0.23, 0.35) |
| New Zealand | 284007 (215830, 363374) | 637031 (494944, 800736) | 10712 (8137.34, 13715.07) | 13732.74 (10640.46, 17303.1) | 0.79 (0.77, 0.81) |
| Niue | 318 (244, 404) | 336 (260, 422) | 21437.76 (16410.87, 27261.27) | 24292.06 (18761.05, 30566.48) | 0.43 (0.42, 0.45) |
| Northern Mariana Islands | 6610 (5066, 8373) | 10309 (7984, 12861) | 24271.4 (18735.74, 30488.44) | 26270.22 (20338.73, 32802.28) | 0.21 (0.16, 0.26) |
| Palau | 1996 (1529, 2537) | 4075 (3145, 5091) | 21660.43 (16636.47, 27434.57) | 24880.24 (19181.57, 31130.98) | 0.44 (0.39, 0.49) |
| Papua New Guinea | 341733 (259996, 439171) | 1103778 (838796, 1407427) | 17632.15 (13459.72, 22605.57) | 19934.36 (15200.11, 25326.96) | 0.44 (0.42, 0.46) |
| Philippines | 5388606 (4122710, 6865231) | 13789125 (10588632, 17466753) | 17383.91 (13345.41, 22094.69) | 18998.78 (14620.06, 24013.54) | 0.3 (0.29, 0.32) |
| Samoa | 17228 (13148, 21849) | 28113 (21527, 35426) | 21733.36 (16637.46, 27468.41) | 23210.95 (17832.64, 29184.48) | 0.16 (0.12, 0.2) |
| Singapore | 349278 (266271, 448825) | 987830 (756259, 1256012) | 15505.71 (11831.45, 19882.03) | 18024.43 (13774.44, 22898.05) | 0.47 (0.44, 0.51) |
| Solomon Islands | 27078 (20650, 34772) | 75901 (58057, 96814) | 18810.3 (14392.62, 24075.32) | 21206.73 (16273.11, 26942.14) | 0.42 (0.36, 0.47) |
| Tokelau | 189 (144, 239) | 233 (180, 294) | 20023.16 (15253.66, 25368.86) | 23259.12 (17918.14, 29374.7) | 0.53 (0.51, 0.56) |
| Tonga | 10890 (8319, 13835) | 15690 (12047, 19774) | 22546.28 (17302.16, 28571.96) | 25087.77 (19294.86, 31577.83) | 0.26 (0.19, 0.33) |
| Tuvalu | 1081 (823, 1379) | 1841 (1418, 2341) | 19378.74 (14749.94, 24670.99) | 22574.31 (17398.71, 28659.39) | 0.51 (0.48, 0.54) |
| Vanuatu | 15149 (11586, 19264) | 44424 (34178, 56073) | 22183.09 (17031.88, 28125.07) | 25894.09 (19973.72, 32605.19) | 0.52 (0.51, 0.53) |
| Viet Nam | 6369338 (4854988, 8140922) | 14621077 (11267209, 18543906) | 17814.51 (13620.32, 22745.2) | 18876.32 (14551.25, 23922.5) | 0.21 (0.19, 0.22) |
| Western Pacific Region | 169857829 (130374539, 215903498) | 359206760 (278424629, 451645724) | 16731.39 (12859.69, 21244.9) | 20431.7 (15787.81, 25742.34) | 0.68 (0.5, 0.87) |

**Table S3. Disability-adjusted life years (DALYs) and age-standardized rate of DALYs (ASR-DALYs) for total MASLD in 31 West Pacific countries in 1990 and 2021, and estimated annual percentage changes (EAPC) of ASR-DALYs cross 1990-2021.**

| **Location** | **1990 DALYs(95%UI)** | **2021 DALYs(95%UI)** | **1990 ASR-DALYs (95% UI)** | **2021 ASR-DALYs (95% UI)** | **EAPC of ASR-DALYs (95% UI)** |
| --- | --- | --- | --- | --- | --- |
| American Samoa | 16 (8, 27) | 36 (20, 58) | 74.61 (40.81, 127.71) | 97.63 (55.69, 158.94) | 0.63 (0.45, 0.81) |
| Australia | 4341 (2621, 6828) | 13746 (9244, 19605) | 32.22 (19.35, 50.81) | 49.37 (32.9, 70.7) | 1.71 (1.57, 1.86) |
| Brunei Darussalam | 36 (21, 60) | 99 (57, 160) | 45.16 (26.17, 75.05) | 36.4 (21.19, 58.47) | -0.74 (-0.85, -0.62) |
| Cambodia | 3573 (1701, 7111) | 7199 (3768, 12723) | 95.42 (44.86, 195.02) | 74.61 (39.14, 131.89) | -1.01 (-1.09, -0.93) |
| China | 253396 (174318, 362576) | 414471 (288295, 577223) | 38.4 (26.45, 55.01) | 27.81 (19.34, 38.78) | -0.97 (-1.11, -0.83) |
| Cook Islands | 4 (2, 8) | 9 (5, 17) | 45.05 (23.24, 79.02) | 52.86 (27.32, 93.25) | 0.47 (0.27, 0.67) |
| Democratic People's Republic of Korea | 4815 (2514, 8619) | 7051 (3688, 12153) | 37.74 (19.6, 68.14) | 29.3 (15.34, 50.61) | -0.86 (-0.92, -0.8) |
| Fiji | 122 (66, 214) | 284 (150, 473) | 35.84 (19.44, 63.14) | 46.09 (24.68, 76.64) | 0.72 (0.66, 0.78) |
| Guam | 45 (25, 79) | 117 (68, 191) | 67.3 (37.39, 115.9) | 83.45 (47.95, 136.4) | 1.23 (1.02, 1.45) |
| Japan | 54593 (38246, 78187) | 53177 (35883, 75782) | 44.84 (31.31, 64.35) | 22.77 (15.3, 33.11) | -2.43 (-2.65, -2.22) |
| Kiribati | 28 (15, 50) | 56 (29, 98) | 83.29 (43.89, 146.09) | 84.48 (44.8, 147.28) | -0.24 (-0.49, 0.02) |
| Lao People's Democratic Republic | 1026 (557, 1787) | 1678 (906, 2869) | 61.88 (33.14, 109.12) | 44.71 (24.4, 76.1) | -1.24 (-1.3, -1.18) |
| Malaysia | 2186 (1305, 3510) | 10320 (6142, 16461) | 30.35 (18.22, 48.59) | 49.28 (29.31, 78.55) | 1.32 (0.97, 1.67) |
| Marshall Islands | 9 (4, 16) | 20 (10, 37) | 55.49 (26.21, 102.23) | 59.75 (29.36, 109.48) | 0.08 (-0.03, 0.19) |
| Micronesia (Federated States of) | 32 (16, 59) | 50 (25, 90) | 75.25 (38.52, 136.55) | 78.21 (39.73, 138.65) | -0.11 (-0.33, 0.11) |
| Mongolia | 1850 (1117, 2933) | 4763 (2890, 7524) | 223 (135.65, 351.76) | 273.86 (167.16, 431.9) | 0.95 (0.6, 1.3) |
| Nauru | 4 (2, 7) | 5 (2, 9) | 89.91 (45.41, 160.67) | 82.86 (35.43, 155.67) | -0.72 (-0.91, -0.53) |
| New Zealand | 623 (418, 899) | 1761 (1292, 2282) | 23.3 (15.54, 33.72) | 32.43 (23.7, 42.19) | 1.04 (0.9, 1.17) |
| Niue | 1 (0, 2) | 1 (1, 2) | 57.89 (30.63, 102.66) | 74.63 (37.38, 130.2) | 0.48 (0.32, 0.64) |
| Northern Mariana Islands | 27 (13, 49) | 50 (29, 82) | 131.08 (70.49, 230.2) | 124.12 (71.24, 201.25) | -0.63 (-0.78, -0.48) |
| Palau | 6 (3, 10) | 15 (8, 27) | 68.28 (35.9, 122.21) | 87.34 (44.9, 153.99) | 0.59 (0.36, 0.82) |
| Papua New Guinea | 364 (154, 797) | 823 (384, 1648) | 22.03 (9.47, 49.08) | 17.22 (8.08, 35.1) | -1.07 (-1.17, -0.98) |
| Philippines | 8948 (5597, 14337) | 24382 (16651, 35090) | 35.41 (21.85, 57.85) | 37.84 (26.04, 54.11) | 0.18 (0.13, 0.23) |
| Samoa | 44 (22, 78) | 69 (36, 120) | 61.47 (31.84, 108.71) | 59.87 (31.5, 103.22) | -0.39 (-0.53, -0.26) |
| Singapore | 477 (290, 747) | 912 (570, 1409) | 28.15 (17.26, 43.71) | 14.92 (9.3, 23.05) | -2.06 (-2.32, -1.79) |
| Solomon Islands | 69 (28, 143) | 219 (113, 395) | 55.41 (23.17, 115.34) | 66.94 (34.83, 119.83) | 0.55 (0.39, 0.71) |
| Tokelau | 0 (0, 1) | 1 (0, 1) | 51.46 (24.9, 96.29) | 58.49 (30.93, 100.56) | 0.1 (-0.04, 0.24) |
| Tonga | 66 (37, 111) | 100 (55, 168) | 150.51 (84.07, 254.77) | 167.19 (91.44, 279.59) | -0.04 (-0.41, 0.34) |
| Tuvalu | 3 (2, 6) | 5 (3, 9) | 61.4 (31.05, 113.35) | 63.32 (34.2, 109.81) | -0.03 (-0.1, 0.04) |
| Vanuatu | 37 (15, 82) | 105 (50, 203) | 62.35 (25.96, 137.93) | 66.08 (31.71, 127.14) | 0.09 (0.01, 0.17) |
| Viet Nam | 17444 (9573, 30235) | 40900 (22534, 69159) | 58.44 (32.09, 101.56) | 54.73 (30.29, 92.07) | -0.09 (-0.18, -0.01) |
| Western Pacific Region | 372034 (260594, 529822) | 600447 (423839, 829044) | 42.14 (29.5, 60.06) | 30.4 (21.41, 42.07) | -1.07 (-1.15, -0.98) |

**Table S4. Incident cases and age-standardized incidence rate (ASIR) for non-malignant MASLD in 31 West Pacific countries in 1990 and 2021, and estimated annual percentage changes (EAPC) of ASIR cross 1990-2021.**

| **Location** | **1990 incident cases(95%UI)** | **2021 incident cases(95%UI)** | **1990 ASIR per 100,000(95% UI)** | **2021 ASIR per 100,000(95% UI)** | **EAPC of ASIR (95% UI)** |
| --- | --- | --- | --- | --- | --- |
| American Samoa | 344 (239, 472) | 411 (281, 573) | 1035.35 (704.56, 1447.21) | 1155.62 (787.06, 1608.87) | 0.34 (0.29, 0.4) |
| Australia | 51514 (35311, 71031) | 96710 (65489, 135552) | 398.17 (273.88, 547.78) | 497.46 (344.11, 684.96) | 0.73 (0.68, 0.77) |
| Brunei Darussalam | 1062 (742, 1437) | 2407 (1634, 3328) | 595.58 (408.99, 818.84) | 685.25 (466.12, 945.14) | 0.47 (0.45, 0.49) |
| Cambodia | 37985 (26448, 51692) | 86497 (59896, 118230) | 647.18 (441.83, 895.68) | 710.83 (488.64, 976.57) | 0.37 (0.3, 0.43) |
| China | 6179750 (4317866, 8402891) | 9561143 (6461232, 13334945) | 694.41 (477.95, 955.23) | 871.15 (601.85, 1192.85) | 0.74 (0.58, 0.91) |
| Cook Islands | 106 (73, 145) | 123 (83, 173) | 799.99 (544.09, 1111.93) | 935.04 (636.56, 1300.7) | 0.58 (0.53, 0.63) |
| Democratic People's Republic of Korea | 99336 (68535, 136498) | 162637 (111079, 225134) | 667.43 (458.04, 921.15) | 766.62 (528.98, 1051.37) | 0.45 (0.44, 0.47) |
| Fiji | 4892 (3435, 6648) | 7556 (5235, 10401) | 956.03 (660.22, 1316.17) | 1137.87 (787.44, 1566.56) | 0.52 (0.48, 0.56) |
| Guam | 816 (567, 1114) | 1081 (740, 1498) | 802.96 (550.77, 1108.25) | 918.28 (634.51, 1263.54) | 0.47 (0.45, 0.5) |
| Japan | 409730 (283038, 565364) | 463951 (311371, 654264) | 413.06 (288.55, 564.61) | 448.83 (313.55, 613.12) | 0.24 (0.18, 0.3) |
| Kiribati | 447 (316, 602) | 879 (617, 1194) | 930.07 (645.09, 1274.3) | 1052.95 (731.19, 1441.63) | 0.33 (0.27, 0.39) |
| Lao People's Democratic Republic | 13938 (9673, 19030) | 34652 (23780, 47377) | 576.31 (393.13, 798.66) | 662.52 (450.04, 914.38) | 0.45 (0.44, 0.47) |
| Malaysia | 109928 (76754, 149388) | 267091 (183120, 369286) | 928.96 (638.33, 1280.7) | 1079.26 (739.11, 1493.8) | 0.5 (0.48, 0.51) |
| Marshall Islands | 194 (137, 262) | 364 (254, 501) | 767.81 (528.56, 1058) | 896.99 (618.74, 1241.8) | 0.47 (0.46, 0.49) |
| Micronesia (Federated States of) | 490 (344, 667) | 707 (491, 969) | 791.67 (542.08, 1096.88) | 932.09 (640.3, 1287.23) | 0.48 (0.42, 0.55) |
| Mongolia | 9474 (6725, 12727) | 17023 (11723, 23242) | 694.1 (480.36, 952.26) | 763.14 (526.11, 1038.72) | 0.36 (0.3, 0.43) |
| Nauru | 50 (35, 68) | 68 (48, 92) | 782.69 (533.64, 1083.18) | 899.89 (621.73, 1236.92) | 0.4 (0.33, 0.46) |
| New Zealand | 11306 (7834, 15529) | 20928 (14232, 28959) | 432.04 (299.65, 593.48) | 527.36 (364.68, 720.47) | 0.68 (0.66, 0.7) |
| Niue | 12 (9, 17) | 12 (8, 17) | 809.67 (558.85, 1128.8) | 969.98 (664.49, 1346.6) | 0.56 (0.53, 0.6) |
| Northern Mariana Islands | 328 (225, 452) | 364 (248, 507) | 915.33 (622.82, 1273.41) | 1017.53 (698.61, 1403.71) | 0.32 (0.26, 0.37) |
| Palau | 95 (66, 129) | 137 (92, 193) | 841.08 (577.31, 1161.14) | 990.67 (678.7, 1365.96) | 0.51 (0.46, 0.56) |
| Papua New Guinea | 17865 (12627, 24137) | 53441 (37674, 72095) | 702.31 (486.41, 965.67) | 777.38 (540.85, 1059.78) | 0.35 (0.33, 0.38) |
| Philippines | 265955 (186788, 359407) | 606429 (420638, 828517) | 671.69 (461.02, 925.71) | 744.85 (512, 1025.35) | 0.33 (0.32, 0.34) |
| Samoa | 903 (637, 1228) | 1286 (901, 1767) | 826.48 (567.41, 1150.59) | 908.5 (628.16, 1259.9) | 0.26 (0.23, 0.29) |
| Singapore | 14644 (10172, 20058) | 29957 (19954, 42554) | 596.6 (411.59, 822.01) | 681.85 (469.65, 942.68) | 0.39 (0.35, 0.42) |
| Solomon Islands | 1453 (1026, 1951) | 3833 (2670, 5197) | 738.12 (508.17, 1011.92) | 854.66 (586.2, 1172.42) | 0.48 (0.45, 0.52) |
| Tokelau | 8 (6, 11) | 9 (6, 12) | 772.58 (530.78, 1057.04) | 924.82 (641.4, 1270.67) | 0.61 (0.58, 0.64) |
| Tonga | 538 (381, 725) | 701 (486, 958) | 855.32 (590.56, 1175.75) | 987.46 (676.11, 1361.16) | 0.4 (0.34, 0.45) |
| Tuvalu | 47 (32, 64) | 81 (56, 111) | 751.87 (519.33, 1034.02) | 908.77 (624.43, 1248.15) | 0.55 (0.5, 0.6) |
| Vanuatu | 799 (563, 1077) | 2132 (1501, 2896) | 893.67 (616.65, 1223.01) | 1027.08 (713.69, 1408.39) | 0.46 (0.45, 0.47) |
| Viet Nam | 284796 (199774, 384207) | 535516 (366662, 739813) | 651.01 (448.36, 891.85) | 718.39 (495.3, 985.91) | 0.46 (0.41, 0.51) |
| Western Pacific Region | 7582244 (5302666, 10297377) | 12022372 (8168174, 16713197) | 655.47 (452.47, 899.37) | 814.16 (564.22, 1113.66) | 0.73 (0.59, 0.86) |

**Table S5. Prevalent cases and age-standardized prevalence rate (ASPR) for non-malignant MASLD in 31 West Pacific countries in 1990 and 2021, and estimated annual percentage changes (EAPC) of ASPR cross 1990-2021.**

| **Location** | **1990 prevalent cases(95%UI)** | **2021 prevalent cases(95%UI)** | **1990 ASPR per 100,000(95% UI)** | **2021 ASPR per 100,000(95% UI)** | **EAPC of ASPR (95% UI)** |
| --- | --- | --- | --- | --- | --- |
| American Samoa | 6059 (4656, 7655) | 10312 (8043, 12741) | 24895.93 (19270.72, 31219.39) | 28873.14 (22519.45, 35702.77) | 0.47 (0.41, 0.53) |
| Australia | 1372025 (1042169, 1760041) | 3141222 (2408177, 3961570) | 10272.02 (7799.54, 13177.88) | 13188.2 (10072.06, 16677.14) | 0.84 (0.81, 0.88) |
| Brunei Darussalam | 19625 (14886, 25139) | 59707 (45593, 75930) | 13553.36 (10293.35, 17362.21) | 17250.97 (13184.7, 21911.58) | 0.91 (0.86, 0.95) |
| Cambodia | 821520 (625683, 1044337) | 1959476 (1499795, 2497386) | 17797.67 (13581.06, 22612.98) | 17863.09 (13680.04, 22736.2) | 0.08 (0.05, 0.1) |
| China | 137113465 (105142399, 174336596) | 291247168 (225495614, 366302163) | 17933.02 (13768.89, 22769.37) | 21886.28 (16887.18, 27587.71) | 0.67 (0.45, 0.9) |
| Cook Islands | 2492 (1916, 3148) | 3763 (2929, 4693) | 22808.42 (17590.17, 28722.12) | 24275.73 (18814.61, 30419.27) | 0.26 (0.17, 0.34) |
| Democratic People's Republic of Korea | 2338263 (1784685, 2987988) | 4521922 (3482019, 5734826) | 16991.3 (12982.24, 21714.57) | 19654.34 (15133.68, 24936.69) | 0.5 (0.47, 0.52) |
| Fiji | 96310 (73777, 122315) | 179992 (139156, 225678) | 23868.19 (18328.76, 30182.01) | 28304.61 (21912.81, 35436.33) | 0.53 (0.48, 0.58) |
| Guam | 17123 (13115, 21817) | 31853 (24811, 39602) | 21204.48 (16318.87, 26856.7) | 23986.34 (18640.49, 29902.27) | 0.44 (0.41, 0.48) |
| Japan | 11268047 (8619714, 14370826) | 16309045 (12599252, 20679433) | 10323.79 (7892.14, 13174.72) | 11405.18 (8780.78, 14465.44) | 0.28 (0.2, 0.35) |
| Kiribati | 8438 (6458, 10753) | 18105 (13905, 22944) | 22461.19 (17230.96, 28509.66) | 25690.33 (19797.19, 32441.87) | 0.35 (0.29, 0.42) |
| Lao People's Democratic Republic | 294393 (224006, 378493) | 745072 (565735, 954971) | 15091.36 (11490.15, 19390.37) | 16614.9 (12647.58, 21254.95) | 0.37 (0.35, 0.39) |
| Malaysia | 2281008 (1737412, 2905185) | 6440024 (4970875, 8107245) | 23608.24 (18054.67, 29987.18) | 27364.96 (21160.7, 34411.99) | 0.48 (0.46, 0.49) |
| Marshall Islands | 3548 (2700, 4551) | 7610 (5821, 9674) | 19505.58 (14891.19, 24928.27) | 22069.97 (16950.58, 27945.81) | 0.41 (0.38, 0.43) |
| Micronesia (Federated States of) | 9394 (7173, 12010) | 15073 (11566, 19107) | 20052.43 (15357.98, 25567.67) | 23134.72 (17834.06, 29225.39) | 0.43 (0.38, 0.48) |
| Mongolia | 189429 (143965, 243458) | 417478 (318860, 532116) | 18153.59 (13838.02, 23266.13) | 19297.96 (14765.63, 24537.68) | 0.31 (0.28, 0.35) |
| Nauru | 983 (751, 1252) | 1287 (985, 1637) | 19846.39 (15206.43, 25209.44) | 21773.05 (16725.94, 27581.06) | 0.29 (0.23, 0.35) |
| New Zealand | 283997 (215820, 363365) | 636948 (494858, 800651) | 10711.64 (8136.94, 13714.72) | 13731.27 (10638.94, 17301.57) | 0.79 (0.77, 0.81) |
| Niue | 318 (244, 404) | 336 (260, 422) | 21437.18 (16410.22, 27260.67) | 24291.18 (18760.1, 30565.57) | 0.43 (0.42, 0.45) |
| Northern Mariana Islands | 6610 (5066, 8373) | 10308 (7984, 12861) | 24270.51 (18734.83, 30487.41) | 26268.89 (20337.42, 32800.91) | 0.21 (0.16, 0.26) |
| Palau | 1996 (1529, 2537) | 4075 (3145, 5091) | 21659.67 (16635.72, 27433.76) | 24879.19 (19180.55, 31129.95) | 0.44 (0.39, 0.49) |
| Papua New Guinea | 341727 (259990, 439166) | 1103766 (838783, 1407414) | 17631.76 (13459.33, 22605.22) | 19934.06 (15199.82, 25326.65) | 0.44 (0.42, 0.46) |
| Philippines | 5388438 (4122550, 6865048) | 13788657 (10588183, 17466267) | 17383.21 (13344.74, 22093.93) | 18998.03 (14619.33, 24012.76) | 0.3 (0.29, 0.32) |
| Samoa | 17227 (13148, 21848) | 28113 (21526, 35425) | 21732.67 (16636.61, 27467.69) | 23210.22 (17831.84, 29183.77) | 0.16 (0.12, 0.2) |
| Singapore | 349268 (266261, 448816) | 987769 (756190, 1255948) | 15505.12 (11830.84, 19881.46) | 18023.42 (13773.31, 22896.98) | 0.47 (0.44, 0.51) |
| Solomon Islands | 27077 (20649, 34771) | 75899 (58055, 96812) | 18809.69 (14391.75, 24074.69) | 21206.15 (16272.38, 26941.44) | 0.42 (0.36, 0.47) |
| Tokelau | 189 (144, 239) | 233 (180, 294) | 20022.64 (15253.1, 25368.42) | 23258.37 (17917.27, 29373.99) | 0.53 (0.51, 0.56) |
| Tonga | 10889 (8318, 13834) | 15688 (12045, 19772) | 22543.52 (17299.51, 28569.14) | 25084.34 (19291.05, 31574.15) | 0.26 (0.19, 0.33) |
| Tuvalu | 1081 (822, 1379) | 1841 (1418, 2341) | 19378.18 (14749.36, 24670.39) | 22573.6 (17397.98, 28658.49) | 0.51 (0.48, 0.54) |
| Vanuatu | 15149 (11586, 19263) | 44423 (34177, 56072) | 22182.57 (17031.31, 28124.54) | 25893.51 (19973.19, 32604.64) | 0.52 (0.51, 0.53) |
| Viet Nam | 6368917 (4854485, 8140470) | 14619894 (11265767, 18542734) | 17813.1 (13618.66, 22743.69) | 18874.74 (14549.32, 23920.93) | 0.21 (0.19, 0.22) |
| Western Pacific Region | 169850180 (130366949, 215896217) | 359184474 (278402564, 451624361) | 16730.5 (12858.81, 21244.06) | 20430.58 (15786.7, 25741.27) | 0.68 (0.5, 0.87) |

**Table S6. Disability-adjusted life years (DALYs) and age-standardized rate of DALYs (ASR-DALYs) for non-malignant MASLD in 31 West Pacific countries in 1990 and 2021, and estimated annual percentage changes (EAPC) of ASR-DALYs cross 1990-2021.**

| **Location** | **1990 DALYs(95%UI)** | **2021 DALYs(95%UI)** | **1990 ASR-DALYs (95% UI)** | **2021 ASR-DALYs (95% UI)** | **EAPC of ASR-DALYs (95% UI)** |
| --- | --- | --- | --- | --- | --- |
| American Samoa | 12 (6, 23) | 24 (11, 43) | 55.22 (25.44, 104.34) | 63.7 (29.94, 115.32) | 0.12 (-0.03, 0.28) |
| Australia | 3640 (1998, 6033) | 8314 (4859, 12757) | 27.13 (14.84, 45.06) | 31.05 (17.93, 48.04) | 0.85 (0.67, 1.02) |
| Brunei Darussalam | 16 (7, 29) | 44 (21, 84) | 16.94 (7.83, 31.76) | 14.99 (6.96, 28.12) | -0.09 (-0.3, 0.13) |
| Cambodia | 2631 (1081, 5731) | 5170 (2279, 10274) | 69.08 (27.95, 154.65) | 52.89 (23.25, 105.89) | -1.09 (-1.17, -1) |
| China | 128243 (65101, 224926) | 158263 (82575, 271498) | 19.62 (10.06, 34.31) | 10.67 (5.53, 18.37) | -2.03 (-2.19, -1.88) |
| Cook Islands | 1 (0, 1) | 1 (0, 2) | 5.48 (2.3, 10.98) | 6.56 (2.68, 13.32) | 0.28 (0.08, 0.48) |
| Democratic People's Republic of Korea | 2079 (906, 4258) | 3027 (1218, 6274) | 16.42 (7.14, 33.74) | 12.61 (5.08, 26.2) | -0.81 (-0.86, -0.76) |
| Fiji | 78 (35, 155) | 161 (70, 312) | 21.4 (9.56, 42.58) | 25.57 (11.2, 49.45) | 0.3 (0.17, 0.43) |
| Guam | 38 (18, 72) | 83 (40, 153) | 55.86 (26.96, 104.14) | 60.31 (28.89, 110.84) | 0.77 (0.52, 1.01) |
| Japan | 33965 (18703, 57376) | 30655 (16680, 51494) | 28.15 (15.5, 47.5) | 14.16 (7.66, 24.01) | -2.14 (-2.3, -1.99) |
| Kiribati | 20 (8, 40) | 39 (17, 78) | 57.75 (23.67, 114.78) | 57.67 (25.07, 114.75) | -0.29 (-0.52, -0.07) |
| Lao People's Democratic Republic | 570 (239, 1178) | 939 (401, 1882) | 33.82 (13.95, 71.36) | 24.38 (10.52, 48.95) | -1.22 (-1.29, -1.15) |
| Malaysia | 1229 (589, 2304) | 5733 (2686, 10766) | 16.52 (8.01, 30.73) | 27.31 (12.78, 51.17) | 1.08 (0.76, 1.39) |
| Marshall Islands | 7 (3, 14) | 15 (6, 32) | 44.64 (18.67, 87.22) | 44.95 (18.22, 91.66) | -0.14 (-0.23, -0.05) |
| Micronesia (Federated States of) | 25 (11, 50) | 37 (16, 75) | 57.62 (24.52, 115.5) | 56.41 (23.75, 112.82) | -0.3 (-0.47, -0.13) |
| Mongolia | 862 (414, 1593) | 1638 (795, 2987) | 103.81 (50.42, 190.26) | 89.92 (44.02, 162.66) | -0.15 (-0.52, 0.22) |
| Nauru | 3 (1, 6) | 3 (1, 7) | 65.93 (26.71, 133.57) | 60.2 (19.81, 127.81) | -0.68 (-0.84, -0.52) |
| New Zealand | 459 (263, 725) | 948 (578, 1381) | 17.25 (9.83, 27.35) | 17.65 (10.65, 25.92) | 0.11 (-0.11, 0.32) |
| Niue | 1 (0, 1) | 1 (0, 1) | 42.42 (18.99, 84.06) | 52.61 (21.79, 102.59) | 0.28 (0.14, 0.43) |
| Northern Mariana Islands | 23 (11, 45) | 38 (19, 67) | 108.18 (51.7, 203.61) | 93.36 (45.44, 165.46) | -0.93 (-1.09, -0.76) |
| Palau | 4 (2, 8) | 10 (4, 20) | 47.64 (19.83, 95.95) | 59.83 (24.87, 119.05) | 0.58 (0.43, 0.72) |
| Papua New Guinea | 204 (80, 427) | 479 (202, 968) | 11.31 (4.52, 23.74) | 9.25 (3.99, 18.59) | -0.92 (-1.06, -0.77) |
| Philippines | 4256 (1933, 8368) | 11927 (6075, 20962) | 16.62 (7.47, 33.16) | 18.26 (9.4, 31.91) | 0.34 (0.29, 0.39) |
| Samoa | 32 (13, 64) | 49 (22, 97) | 43.18 (18.03, 86.26) | 41.41 (18.4, 81.33) | -0.42 (-0.56, -0.29) |
| Singapore | 284 (137, 516) | 358 (183, 626) | 16.15 (7.88, 29.16) | 5.87 (2.98, 10.3) | -3.33 (-3.5, -3.16) |
| Solomon Islands | 49 (18, 106) | 171 (77, 335) | 38.27 (14.65, 82.18) | 51.02 (23.15, 99.13) | 0.89 (0.76, 1.03) |
| Tokelau | 0 (0, 1) | 0 (0, 1) | 37.65 (15.77, 75.74) | 39.64 (17.47, 76.13) | -0.15 (-0.28, -0.02) |
| Tonga | 34 (16, 66) | 49 (21, 95) | 77.03 (34.86, 146.67) | 80.14 (34.29, 155.65) | -0.2 (-0.46, 0.05) |
| Tuvalu | 3 (1, 5) | 4 (2, 7) | 45.94 (19.29, 93.1) | 44.66 (20.1, 87.27) | -0.22 (-0.29, -0.15) |
| Vanuatu | 30 (11, 70) | 84 (33, 176) | 48.21 (16.93, 114.06) | 50.61 (19.88, 106.41) | 0 (-0.08, 0.09) |
| Viet Nam | 6196 (2579, 12944) | 13353 (5743, 26237) | 21.11 (8.78, 44.38) | 18.36 (7.93, 35.97) | -0.38 (-0.52, -0.24) |
| Western Pacific Region | 190154 (98311, 330658) | 243719 (129572, 412356) | 21.78 (11.36, 37.73) | 12.45 (6.57, 21.12) | -1.84 (-1.98, -1.71) |

**Table S7. Deaths for total and non-malignant MASLD in 31 West Pacific countries in 1990 and 2021.**

| **Location** | **Total MASLD** | | **Non-malignant MASLD** |  |
| --- | --- | --- | --- | --- |
|  | **1990 deaths(95%UI)** | **2021 deaths(95%UI)** | **1990 deaths(95%UI)** | **2021 deaths(95%UI)** |
| American Samoa | 0 (0, 1) | 1 (1, 2) | 0 (0, 1) | 1 (0, 1) |
| Australia | 157 (97, 242) | 581 (395, 826) | 127 (71, 209) | 321 (191, 484) |
| Brunei Darussalam | 1 (1, 2) | 4 (2, 6) | 0 (0, 1) | 1 (1, 3) |
| Cambodia | 115 (54, 236) | 250 (132, 441) | 83 (33, 186) | 175 (77, 351) |
| China | 8351 (5785, 11914) | 16748 (11708, 23103) | 4223 (2188, 7372) | 6339 (3343, 10806) |
| Cook Islands | 0 (0, 0) | 0 (0, 1) | 0 (0, 0) | 0 (0, 0) |
| Democratic People's Republic of Korea | 159 (83, 288) | 258 (137, 449) | 69 (30, 142) | 110 (45, 229) |
| Fiji | 3 (2, 6) | 9 (5, 15) | 2 (1, 4) | 5 (2, 9) |
| Guam | 1 (1, 2) | 4 (2, 6) | 1 (1, 2) | 3 (1, 5) |
| Japan | 2248 (1571, 3198) | 3274 (2167, 4650) | 1410 (785, 2346) | 1803 (969, 3027) |
| Kiribati | 1 (0, 1) | 2 (1, 3) | 1 (0, 1) | 1 (0, 2) |
| Lao People's Democratic Republic | 33 (18, 59) | 57 (31, 96) | 18 (7, 38) | 30 (13, 61) |
| Malaysia | 78 (47, 126) | 403 (241, 641) | 42 (20, 78) | 221 (104, 412) |
| Marshall Islands | 0 (0, 0) | 1 (0, 1) | 0 (0, 0) | 0 (0, 1) |
| Micronesia (Federated States of) | 1 (0, 2) | 1 (1, 3) | 1 (0, 1) | 1 (0, 2) |
| Mongolia | 64 (39, 101) | 174 (106, 275) | 30 (15, 55) | 57 (28, 102) |
| Nauru | 0 (0, 0) | 0 (0, 0) | 0 (0, 0) | 0 (0, 0) |
| New Zealand | 24 (16, 33) | 75 (55, 97) | 17 (10, 26) | 38 (24, 55) |
| Niue | 0 (0, 0) | 0 (0, 0) | 0 (0, 0) | 0 (0, 0) |
| Northern Mariana Islands | 1 (0, 1) | 2 (1, 3) | 1 (0, 1) | 1 (1, 2) |
| Palau | 0 (0, 0) | 0 (0, 1) | 0 (0, 0) | 0 (0, 1) |
| Papua New Guinea | 10 (4, 23) | 24 (11, 48) | 5 (2, 11) | 13 (5, 26) |
| Philippines | 282 (173, 462) | 846 (587, 1202) | 131 (59, 263) | 402 (209, 699) |
| Samoa | 1 (1, 2) | 2 (1, 4) | 1 (0, 2) | 1 (1, 3) |
| Singapore | 18 (11, 27) | 43 (27, 66) | 10 (5, 18) | 16 (8, 27) |
| Solomon Islands | 2 (1, 4) | 6 (3, 11) | 1 (1, 3) | 5 (2, 9) |
| Tokelau | 0 (0, 0) | 0 (0, 0) | 0 (0, 0) | 0 (0, 0) |
| Tonga | 2 (1, 4) | 4 (2, 6) | 1 (0, 2) | 2 (1, 3) |
| Tuvalu | 0 (0, 0) | 0 (0, 0) | 0 (0, 0) | 0 (0, 0) |
| Vanuatu | 1 (0, 2) | 3 (1, 6) | 1 (0, 2) | 2 (1, 5) |
| Viet Nam | 659 (360, 1155) | 1541 (855, 2583) | 243 (99, 520) | 521 (227, 1018) |
| Western Pacific Region | 12887 (9068, 18298) | 25278 (17847, 34611) | 6591 (3475, 11376) | 10195 (5475, 17120) |

**Table S8. Years lived with disability (YLDs) and age-standardized rate of YLDs (ASR-YLDs) for total and non-malignant MASLD in 31 West Pacific countries in 1990 and 2021, and estimated annual percentage changes (EAPC) of ASR-YLDs cross 1990-2021.**

| **Location** | **Total MASLD** | | | | | **Non-malignant MASLD** | | | | |
| --- | --- | --- | --- | --- | --- | --- | --- | --- | --- | --- |
|  | **1990 YLDs**  **(95%UI)** | **2021 YLDs**  **(95%UI)** | **1990 ASR-YLDs (95% UI)** | **2021 ASR-YLDs (95% UI)** | **EAPC of ASR-YLDs (95% UI)** | **1990 YLDs**  **(95%UI)** | **2021 YLDs**  **(95%UI)** | **1990 ASR-YLDs (95% UI)** | **2021 ASR-YLDs (95% UI)** | **EAPC of ASR-YLDs (95% UI)** |
| American Samoa | 0 (0, 0) | 0 (0, 1) | 0.68 (0.35, 1.21) | 0.96 (0.51, 1.65) | 0.81 (0.58, 1.04) | 0 (0, 0) | 0 (0, 0) | 0.52 (0.24, 0.99) | 0.67 (0.31, 1.24) | 0.45 (0.26, 0.63) |
| Australia | 59 (31, 102) | 225 (130, 359) | 0.44 (0.23, 0.75) | 0.79 (0.45, 1.27) | 2.22 (2.14, 2.31) | 52 (25, 93) | 159 (82, 271) | 0.39 (0.19, 0.69) | 0.57 (0.29, 0.99) | 1.54 (1.46, 1.63) |
| Brunei Darussalam | 0 (0, 1) | 1 (1, 2) | 0.5 (0.26, 0.89) | 0.45 (0.24, 0.79) | -0.5 (-0.56, -0.43) | 0 (0, 0) | 1 (0, 1) | 0.24 (0.1, 0.47) | 0.24 (0.11, 0.45) | -0.17 (-0.31, -0.04) |
| Cambodia | 17 (8, 32) | 45 (23, 83) | 0.46 (0.22, 0.88) | 0.49 (0.25, 0.89) | 0.12 (0.01, 0.24) | 10 (4, 20) | 29 (12, 56) | 0.25 (0.11, 0.49) | 0.29 (0.13, 0.57) | 0.67 (0.5, 0.83) |
| China | 2629 (1473, 4529) | 5931 (3494, 9679) | 0.41 (0.23, 0.69) | 0.41 (0.24, 0.67) | 0.17 (-0.01, 0.35) | 1686 (703, 3447) | 3239 (1428, 6368) | 0.25 (0.11, 0.51) | 0.23 (0.1, 0.44) | -0.33 (-0.56, -0.11) |
| Cook Islands | 0 (0, 0) | 0 (0, 0) | 0.59 (0.31, 1.02) | 0.82 (0.43, 1.41) | 0.88 (0.68, 1.08) | 0 (0, 0) | 0 (0, 0) | 0.25 (0.11, 0.48) | 0.38 (0.17, 0.72) | 1.11 (0.97, 1.25) |
| Democratic People's Republic of Korea | 43 (21, 80) | 73 (38, 130) | 0.35 (0.17, 0.65) | 0.31 (0.16, 0.55) | -0.45 (-0.48, -0.41) | 23 (9, 45) | 39 (17, 78) | 0.18 (0.08, 0.36) | 0.17 (0.07, 0.33) | -0.25 (-0.29, -0.21) |
| Fiji | 1 (0, 2) | 3 (1, 5) | 0.31 (0.16, 0.55) | 0.46 (0.24, 0.81) | 1.14 (1.06, 1.22) | 1 (0, 1) | 2 (1, 3) | 0.19 (0.08, 0.37) | 0.29 (0.13, 0.55) | 1.03 (0.88, 1.18) |
| Guam | 0 (0, 1) | 1 (1, 2) | 0.62 (0.31, 1.11) | 0.88 (0.45, 1.54) | 1.48 (1.36, 1.6) | 0 (0, 1) | 1 (0, 2) | 0.52 (0.23, 0.99) | 0.68 (0.31, 1.27) | 1.19 (1.08, 1.3) |
| Japan | 999 (536, 1754) | 1446 (823, 2363) | 0.83 (0.45, 1.46) | 0.62 (0.34, 1.05) | -1.03 (-1.24, -0.81) | 742 (327, 1464) | 950 (435, 1778) | 0.62 (0.28, 1.23) | 0.45 (0.2, 0.86) | -0.83 (-0.98, -0.67) |
| Kiribati | 0 (0, 0) | 0 (0, 1) | 0.62 (0.32, 1.12) | 0.66 (0.34, 1.17) | -0.22 (-0.49, 0.05) | 0 (0, 0) | 0 (0, 1) | 0.42 (0.18, 0.84) | 0.44 (0.19, 0.88) | -0.3 (-0.55, -0.05) |
| Lao People's Democratic Republic | 6 (3, 10) | 12 (6, 21) | 0.37 (0.19, 0.66) | 0.33 (0.17, 0.58) | -0.49 (-0.54, -0.43) | 2 (1, 5) | 6 (3, 12) | 0.14 (0.06, 0.27) | 0.15 (0.07, 0.3) | 0.38 (0.28, 0.48) |
| Malaysia | 17 (9, 30) | 99 (52, 171) | 0.25 (0.13, 0.42) | 0.49 (0.26, 0.83) | 2.02 (1.68, 2.36) | 9 (4, 19) | 58 (26, 111) | 0.12 (0.05, 0.24) | 0.28 (0.13, 0.53) | 2.21 (1.98, 2.44) |
| Marshall Islands | 0 (0, 0) | 0 (0, 0) | 0.35 (0.17, 0.65) | 0.46 (0.23, 0.82) | 0.62 (0.52, 0.72) | 0 (0, 0) | 0 (0, 0) | 0.26 (0.11, 0.53) | 0.34 (0.14, 0.66) | 0.53 (0.45, 0.61) |
| Micronesia (Federated States of) | 0 (0, 0) | 0 (0, 1) | 0.54 (0.27, 0.99) | 0.71 (0.36, 1.27) | 0.66 (0.45, 0.87) | 0 (0, 0) | 0 (0, 1) | 0.4 (0.17, 0.8) | 0.53 (0.23, 1.03) | 0.7 (0.55, 0.86) |
| Mongolia | 14 (7, 24) | 48 (25, 84) | 1.75 (0.94, 3) | 2.97 (1.54, 5.13) | 2.04 (1.76, 2.31) | 7 (3, 13) | 24 (10, 48) | 0.79 (0.34, 1.54) | 1.39 (0.56, 2.77) | 2.05 (1.93, 2.18) |
| Nauru | 0 (0, 0) | 0 (0, 0) | 0.55 (0.28, 0.99) | 0.56 (0.28, 1) | -0.43 (-0.77, -0.09) | 0 (0, 0) | 0 (0, 0) | 0.36 (0.16, 0.73) | 0.4 (0.17, 0.78) | -0.13 (-0.48, 0.23) |
| New Zealand | 12 (6, 22) | 35 (21, 56) | 0.45 (0.23, 0.8) | 0.63 (0.37, 1) | 1.03 (0.97, 1.1) | 10 (5, 20) | 23 (11, 40) | 0.38 (0.17, 0.73) | 0.41 (0.2, 0.73) | 0.19 (0.13, 0.24) |
| Niue | 0 (0, 0) | 0 (0, 0) | 0.52 (0.26, 0.94) | 0.77 (0.4, 1.35) | 1.06 (0.91, 1.22) | 0 (0, 0) | 0 (0, 0) | 0.4 (0.17, 0.77) | 0.58 (0.26, 1.1) | 1.05 (0.92, 1.17) |
| Northern Mariana Islands | 0 (0, 0) | 1 (0, 1) | 1.2 (0.6, 2.18) | 1.37 (0.7, 2.38) | 0.01 (-0.24, 0.25) | 0 (0, 0) | 0 (0, 1) | 1 (0.45, 1.92) | 1.09 (0.49, 2.02) | -0.13 (-0.41, 0.16) |
| Palau | 0 (0, 0) | 0 (0, 0) | 0.68 (0.33, 1.24) | 0.94 (0.47, 1.69) | 0.76 (0.5, 1.02) | 0 (0, 0) | 0 (0, 0) | 0.51 (0.22, 1.01) | 0.72 (0.32, 1.37) | 0.82 (0.61, 1.03) |
| Papua New Guinea | 3 (1, 6) | 7 (3, 15) | 0.17 (0.07, 0.37) | 0.15 (0.07, 0.32) | -0.61 (-0.73, -0.49) | 2 (1, 3) | 5 (2, 9) | 0.08 (0.03, 0.16) | 0.09 (0.04, 0.17) | -0.04 (-0.15, 0.07) |
| Philippines | 72 (41, 123) | 199 (116, 325) | 0.3 (0.17, 0.5) | 0.32 (0.19, 0.52) | 0.17 (0.1, 0.24) | 38 (16, 79) | 101 (43, 203) | 0.14 (0.06, 0.29) | 0.16 (0.07, 0.31) | 0.11 (0.01, 0.21) |
| Samoa | 0 (0, 1) | 1 (0, 1) | 0.6 (0.3, 1.08) | 0.64 (0.33, 1.14) | -0.09 (-0.27, 0.09) | 0 (0, 1) | 1 (0, 1) | 0.44 (0.19, 0.86) | 0.48 (0.21, 0.93) | -0.02 (-0.2, 0.15) |
| Singapore | 6 (3, 11) | 23 (13, 40) | 0.37 (0.2, 0.64) | 0.4 (0.21, 0.68) | 0.15 (0, 0.3) | 4 (2, 9) | 14 (6, 27) | 0.25 (0.11, 0.48) | 0.25 (0.11, 0.47) | -0.21 (-0.38, -0.04) |
| Solomon Islands | 1 (0, 1) | 2 (1, 4) | 0.43 (0.2, 0.84) | 0.59 (0.29, 1.08) | 0.9 (0.73, 1.07) | 0 (0, 1) | 2 (1, 3) | 0.29 (0.12, 0.58) | 0.46 (0.2, 0.9) | 1.37 (1.22, 1.52) |
| Tokelau | 0 (0, 0) | 0 (0, 0) | 0.4 (0.2, 0.73) | 0.55 (0.28, 0.99) | 0.84 (0.71, 0.97) | 0 (0, 0) | 0 (0, 0) | 0.28 (0.12, 0.56) | 0.39 (0.18, 0.75) | 0.94 (0.83, 1.05) |
| Tonga | 0 (0, 1) | 1 (0, 1) | 1.13 (0.58, 2) | 1.42 (0.76, 2.44) | 0.29 (-0.08, 0.66) | 0 (0, 0) | 0 (0, 1) | 0.52 (0.23, 1.02) | 0.68 (0.31, 1.3) | 0.42 (0.16, 0.67) |
| Tuvalu | 0 (0, 0) | 0 (0, 0) | 0.42 (0.21, 0.76) | 0.56 (0.28, 0.99) | 0.73 (0.63, 0.83) | 0 (0, 0) | 0 (0, 0) | 0.29 (0.12, 0.58) | 0.4 (0.18, 0.78) | 0.83 (0.73, 0.93) |
| Vanuatu | 0 (0, 0) | 1 (0, 1) | 0.4 (0.2, 0.75) | 0.48 (0.24, 0.87) | 0.53 (0.51, 0.56) | 0 (0, 0) | 1 (0, 1) | 0.28 (0.12, 0.58) | 0.35 (0.15, 0.68) | 0.57 (0.51, 0.62) |
| Viet Nam | 139 (70, 252) | 382 (196, 689) | 0.47 (0.24, 0.85) | 0.53 (0.27, 0.95) | 0.51 (0.39, 0.63) | 48 (20, 95) | 144 (63, 282) | 0.16 (0.07, 0.31) | 0.2 (0.09, 0.4) | 0.89 (0.59, 1.19) |
| Western Pacific Region | 4187 (2376, 7141) | 8926 (5314, 14344) | 0.49 (0.28, 0.83) | 0.46 (0.27, 0.74) | -0.19 (-0.27, -0.1) | 2688 (1157, 5370) | 4877 (2188, 9390) | 0.31 (0.14, 0.62) | 0.25 (0.11, 0.49) | -0.61 (-0.77, -0.46) |

**Table S9. Years of life lost (YLLs) and age-standardized rate of YLLs (ASR-YLLs) for total and non-malignant MASLD in 31 West Pacific countries in 1990 and 2021, and estimated annual percentage changes (EAPC) of ASR-YLLs cross 1990-2021.**

| **Location** | **Total MASLD** | | | | | **Non-malignant MASLD** | | | | |
| --- | --- | --- | --- | --- | --- | --- | --- | --- | --- | --- |
|  | **1990 YLLs**  **(95%UI)** | **2021 YLLs**  **(95%UI)** | **1990 ASR-YLLs (95% UI)** | **2021 ASR-YLLs (95% UI)** | **EAPC of ASR-YLLs (95% UI)** | **1990 YLLs**  **(95%UI)** | **2021 YLLs**  **(95%UI)** | **1990 ASR-YLLs (95% UI)** | **2021 ASR-YLLs (95% UI)** | **EAPC of ASR-YLLs (95% UI)** |
| American Samoa | 16 (8, 27) | 36 (20, 58) | 73.93 (40.35, 126.75) | 96.67 (55.01, 157.54) | 0.62 (0.45, 0.8) | 12 (5, 23) | 23 (11, 42) | 54.7 (25.14, 103.53) | 63.03 (29.51, 114.35) | 0.12 (-0.03, 0.27) |
| Australia | 4282 (2584, 6742) | 13521 (9094, 19304) | 31.78 (19.07, 50.16) | 48.58 (32.38, 69.64) | 1.7 (1.56, 1.85) | 3588 (1967, 5954) | 8155 (4762, 12535) | 26.74 (14.61, 44.47) | 30.48 (17.59, 47.23) | 0.83 (0.66, 1.01) |
| Brunei Darussalam | 36 (21, 59) | 98 (57, 158) | 44.66 (25.84, 74.23) | 35.94 (20.91, 57.83) | -0.74 (-0.86, -0.63) | 15 (7, 29) | 44 (20, 83) | 16.7 (7.7, 31.35) | 14.75 (6.81, 27.73) | -0.09 (-0.31, 0.13) |
| Cambodia | 3556 (1691, 7084) | 7154 (3739, 12656) | 94.96 (44.58, 194.26) | 74.12 (38.83, 131.18) | -1.02 (-1.1, -0.94) | 2621 (1075, 5717) | 5141 (2264, 10231) | 68.83 (27.81, 154.29) | 52.6 (23.09, 105.45) | -1.09 (-1.18, -1.01) |
| China | 250767 (172483, 358207) | 408540 (284084, 568953) | 37.99 (26.17, 54.36) | 27.4 (19.05, 38.21) | -0.99 (-1.13, -0.85) | 126558 (64187, 222144) | 155024 (80696, 266589) | 19.36 (9.92, 33.9) | 10.45 (5.4, 18.02) | -2.06 (-2.21, -1.91) |
| Cook Islands | 4 (2, 8) | 9 (5, 16) | 44.46 (22.86, 78.16) | 52.05 (26.77, 91.99) | 0.46 (0.27, 0.66) | 1 (0, 1) | 1 (0, 2) | 5.23 (2.16, 10.58) | 6.18 (2.47, 12.7) | 0.23 (0.02, 0.44) |
| Democratic People's Republic of Korea | 4772 (2488, 8554) | 6979 (3642, 12039) | 37.39 (19.39, 67.6) | 28.99 (15.14, 50.12) | -0.87 (-0.92, -0.81) | 2057 (894, 4224) | 2988 (1196, 6212) | 16.24 (7.04, 33.47) | 12.44 (4.98, 25.93) | -0.82 (-0.87, -0.77) |
| Fiji | 121 (65, 213) | 281 (149, 469) | 35.53 (19.24, 62.66) | 45.62 (24.39, 75.94) | 0.72 (0.66, 0.78) | 78 (35, 154) | 159 (69, 310) | 21.21 (9.45, 42.28) | 25.28 (11.01, 49) | 0.29 (0.16, 0.42) |
| Guam | 45 (24, 79) | 116 (67, 189) | 66.68 (37.03, 114.98) | 82.57 (47.38, 135.1) | 1.23 (1.01, 1.45) | 38 (18, 72) | 83 (40, 151) | 55.34 (26.7, 103.38) | 59.62 (28.49, 109.74) | 0.77 (0.52, 1.01) |
| Japan | 53594 (37528, 76642) | 51731 (34809, 73809) | 44.01 (30.72, 63.06) | 22.15 (14.87, 32.22) | -2.47 (-2.69, -2.25) | 33223 (18301, 56075) | 29705 (16101, 49885) | 27.52 (15.17, 46.4) | 13.71 (7.4, 23.24) | -2.18 (-2.33, -2.03) |
| Kiribati | 28 (15, 49) | 55 (29, 98) | 82.67 (43.49, 145.2) | 83.82 (44.37, 146.32) | -0.24 (-0.49, 0.02) | 20 (8, 40) | 39 (17, 78) | 57.33 (23.39, 114.19) | 57.22 (24.78, 113.99) | -0.29 (-0.52, -0.07) |
| Lao People's Democratic Republic | 1020 (553, 1778) | 1666 (899, 2851) | 61.51 (32.92, 108.53) | 44.38 (24.21, 75.6) | -1.25 (-1.31, -1.19) | 568 (238, 1175) | 933 (398, 1873) | 33.68 (13.87, 71.16) | 24.23 (10.43, 48.71) | -1.22 (-1.3, -1.15) |
| Malaysia | 2169 (1294, 3484) | 10221 (6080, 16308) | 30.11 (18.05, 48.22) | 48.79 (29.01, 77.8) | 1.31 (0.96, 1.67) | 1219 (584, 2289) | 5675 (2655, 10676) | 16.39 (7.94, 30.54) | 27.03 (12.63, 50.75) | 1.07 (0.75, 1.38) |
| Marshall Islands | 9 (4, 16) | 20 (9, 36) | 55.14 (26.02, 101.73) | 59.29 (29.09, 108.81) | 0.08 (-0.03, 0.19) | 7 (3, 14) | 15 (6, 31) | 44.38 (18.52, 86.81) | 44.61 (18, 91.18) | -0.14 (-0.23, -0.05) |
| Micronesia (Federated States of) | 32 (16, 58) | 50 (25, 89) | 74.7 (38.2, 135.79) | 77.5 (39.23, 137.61) | -0.11 (-0.33, 0.1) | 25 (10, 50) | 37 (15, 74) | 57.21 (24.29, 114.92) | 55.88 (23.44, 112.06) | -0.31 (-0.48, -0.14) |
| Mongolia | 1836 (1109, 2911) | 4715 (2858, 7455) | 221.25 (134.56, 349.11) | 270.89 (165.25, 427.59) | 0.94 (0.59, 1.29) | 855 (411, 1583) | 1614 (780, 2949) | 103.02 (49.98, 189.04) | 88.53 (43.13, 160.41) | -0.17 (-0.55, 0.2) |
| Nauru | 4 (2, 7) | 5 (2, 9) | 89.36 (45.06, 159.81) | 82.3 (34.99, 154.89) | -0.72 (-0.91, -0.53) | 3 (1, 6) | 3 (1, 7) | 65.56 (26.48, 133.07) | 59.8 (19.58, 127.13) | -0.68 (-0.84, -0.53) |
| New Zealand | 611 (410, 882) | 1725 (1265, 2238) | 22.85 (15.25, 33.07) | 31.8 (23.23, 41.4) | 1.04 (0.9, 1.17) | 449 (256, 709) | 925 (563, 1350) | 16.87 (9.59, 26.75) | 17.24 (10.39, 25.34) | 0.11 (-0.11, 0.33) |
| Niue | 1 (0, 2) | 1 (1, 2) | 57.36 (30.32, 101.83) | 73.86 (36.84, 129.06) | 0.48 (0.31, 0.64) | 1 (0, 1) | 1 (0, 1) | 42.02 (18.76, 83.42) | 52.03 (21.44, 101.76) | 0.28 (0.13, 0.42) |
| Northern Mariana Islands | 27 (13, 49) | 50 (29, 81) | 129.88 (69.73, 228.38) | 122.75 (70.39, 199.33) | -0.64 (-0.79, -0.49) | 23 (10, 45) | 38 (18, 67) | 107.17 (51.11, 202.08) | 92.27 (44.76, 163.8) | -0.94 (-1.1, -0.77) |
| Palau | 6 (3, 10) | 15 (8, 26) | 67.6 (35.47, 121.26) | 86.4 (44.26, 152.54) | 0.59 (0.36, 0.82) | 4 (2, 8) | 10 (4, 20) | 47.13 (19.53, 95.16) | 59.11 (24.42, 118.01) | 0.57 (0.43, 0.72) |
| Papua New Guinea | 361 (152, 792) | 816 (380, 1637) | 21.86 (9.38, 48.73) | 17.06 (8, 34.84) | -1.08 (-1.17, -0.98) | 202 (79, 425) | 475 (200, 960) | 11.23 (4.48, 23.61) | 9.16 (3.94, 18.44) | -0.92 (-1.07, -0.78) |
| Philippines | 8876 (5550, 14240) | 24183 (16507, 34816) | 35.12 (21.65, 57.46) | 37.52 (25.81, 53.67) | 0.18 (0.13, 0.23) | 4218 (1912, 8304) | 11826 (6020, 20790) | 16.48 (7.39, 32.93) | 18.11 (9.31, 31.65) | 0.34 (0.29, 0.39) |
| Samoa | 43 (22, 77) | 68 (35, 119) | 60.88 (31.45, 107.84) | 59.22 (31.08, 102.2) | -0.4 (-0.53, -0.26) | 31 (13, 63) | 48 (21, 96) | 42.74 (17.77, 85.62) | 40.93 (18.12, 80.55) | -0.43 (-0.56, -0.29) |
| Singapore | 471 (286, 737) | 888 (556, 1376) | 27.78 (17.05, 43.13) | 14.52 (9.07, 22.47) | -2.1 (-2.37, -1.83) | 280 (135, 508) | 344 (175, 601) | 15.9 (7.76, 28.72) | 5.63 (2.85, 9.87) | -3.41 (-3.58, -3.23) |
| Solomon Islands | 68 (28, 142) | 217 (111, 392) | 54.98 (22.92, 114.64) | 66.35 (34.47, 118.92) | 0.55 (0.39, 0.71) | 49 (18, 105) | 169 (76, 333) | 37.98 (14.49, 81.73) | 50.55 (22.9, 98.39) | 0.89 (0.75, 1.02) |
| Tokelau | 0 (0, 1) | 1 (0, 1) | 51.06 (24.65, 95.64) | 57.93 (30.58, 99.68) | 0.09 (-0.05, 0.23) | 0 (0, 1) | 0 (0, 1) | 37.36 (15.61, 75.26) | 39.25 (17.24, 75.57) | -0.16 (-0.29, -0.03) |
| Tonga | 65 (36, 111) | 100 (54, 167) | 149.38 (83.39, 253.05) | 165.77 (90.57, 277.48) | -0.04 (-0.41, 0.33) | 34 (15, 65) | 48 (21, 94) | 76.51 (34.59, 145.79) | 79.45 (33.87, 154.57) | -0.21 (-0.47, 0.05) |
| Tuvalu | 3 (2, 6) | 5 (3, 9) | 60.98 (30.8, 112.66) | 62.76 (33.86, 109.06) | -0.04 (-0.1, 0.03) | 3 (1, 5) | 4 (2, 7) | 45.65 (19.12, 92.64) | 44.26 (19.87, 86.62) | -0.23 (-0.3, -0.16) |
| Vanuatu | 37 (15, 82) | 104 (49, 202) | 61.95 (25.71, 137.36) | 65.6 (31.42, 126.52) | 0.09 (0.01, 0.17) | 30 (10, 70) | 83 (33, 175) | 47.93 (16.74, 113.61) | 50.26 (19.67, 105.88) | 0 (-0.08, 0.08) |
| Viet Nam | 17306 (9492, 30005) | 40517 (22305, 68532) | 57.98 (31.82, 100.79) | 54.19 (29.97, 91.2) | -0.1 (-0.18, -0.02) | 6148 (2553, 12867) | 13209 (5661, 26013) | 20.95 (8.69, 44.13) | 18.16 (7.81, 35.65) | -0.39 (-0.53, -0.25) |
| Western Pacific Region | 367847 (257739, 524191) | 591521 (417292, 816719) | 41.65 (29.16, 59.4) | 29.94 (21.08, 41.44) | -1.08 (-1.16, -1) | 187466 (96802, 326344) | 238841 (126566, 404736) | 21.47 (11.18, 37.24) | 12.19 (6.41, 20.72) | -1.87 (-2, -1.73) |

**Table S10. Regional burdens of total and non-malignant MASLD by genders in 2021.**

|  | **Total MASLD** | | **Non-malignant MASLD** | |
| --- | --- | --- | --- | --- |
| **2021 ASR per 100,000(95%UI)** | **Male** | **Female** | **Male** | **Female** |
| Incidence | 789.77 (551.42, 1076.36) | 836.64 (572.88, 1151.1) | 788.82 (550.45, 1075.38) | 835.92 (572.19, 1150.38) |
| Prevalence | 20459.8 (15793.49, 25756.47) | 20356.84 (15674.78, 25788.1) | 20458.51 (15792.23, 25755.27) | 20355.88 (15673.84, 25787.13) |
| Deaths | 1.39 (0.94, 1.96) | 1.16 (0.79, 1.62) | 0.53 (0.28, 0.91) | 0.5 (0.26, 0.85) |
| DALYs | 34.68 (23.4, 49.56) | 26.04 (18.02, 36.4) | 13.77 (7.05, 24.1) | 10.95 (5.78, 18.86) |
| YLLs | 0.45 (0.27, 0.72) | 0.46 (0.26, 0.75) | 13.55 (6.92, 23.72) | 10.67 (5.61, 18.39) |
| YLDs | 34.23 (23.07, 48.96) | 25.58 (17.69, 35.75) | 0.22 (0.09, 0.43) | 0.28 (0.13, 0.54) |

**Table S11. Regional burden of total and non-malignant MASLD by genders and ages in 2021**

| **Age groups** | **Total MASLD** | | | | **Non-malignant MASLD** | | | |
| --- | --- | --- | --- | --- | --- | --- | --- | --- |
|  | **ASIR per 100,000(95%UI)** | **ASPR per 100,000(95%UI)** | **ASDR per 100,000(95%UI)** | **ASR of DALYs per 100,000(95%UI)** | **ASIR per 100,000(95%UI)** | **ASPR per 100,000(95%UI)** | **ASDR per 100,000(95%UI)** | **ASR of DALYs per 100,000(95%UI)** |
| **Female** | | | | | | | | |
| 15 to 19 | 808.95 (612.19, 1034.31) | 3812.59 (2912.41, 4906.2) | 0.02 (0.01, 0.03) | 1.47 (1.04, 1.99) | 808.93 (612.17, 1034.29) | 3812.55 (2912.37, 4906.16) | 0.01 (0, 0.01) | 0.43 (0.17, 0.85) |
| 20 to 24 | 1021.04 (768.32, 1287.8) | 8261.59 (6166.12, 10472.87) | 0.04 (0.02, 0.05) | 2.45 (1.73, 3.35) | 1021.01 (768.3, 1287.77) | 8261.54 (6166.06, 10472.82) | 0.01 (0.01, 0.02) | 0.97 (0.53, 1.69) |
| 25 to 29 | 957.68 (656.14, 1290.3) | 13894.18 (10770.67, 17805.81) | 0.05 (0.03, 0.07) | 3.25 (2.18, 4.56) | 957.64 (656.11, 1290.26) | 13894.07 (10770.58, 17805.71) | 0.02 (0.01, 0.04) | 1.34 (0.61, 2.46) |
| 30 to 34 | 873.38 (608.48, 1196.98) | 17605.43 (13397.78, 22520.9) | 0.08 (0.06, 0.11) | 4.78 (3.3, 6.69) | 873.32 (608.4, 1196.93) | 17605.27 (13397.66, 22520.71) | 0.03 (0.02, 0.06) | 2.01 (1.08, 3.34) |
| 35 to 39 | 808.19 (538.75, 1177.21) | 19275.04 (14663.7, 24779.28) | 0.13 (0.09, 0.19) | 7.16 (4.64, 10.39) | 808.08 (538.63, 1177.14) | 19274.83 (14663.49, 24779.1) | 0.06 (0.02, 0.1) | 3.14 (1.33, 5.56) |
| 40 to 44 | 777.27 (517.48, 1086.11) | 21709.09 (16701.86, 27997.87) | 0.26 (0.17, 0.37) | 12.57 (8.3, 17.89) | 777.08 (517.29, 1085.9) | 21708.74 (16701.55, 27997.55) | 0.12 (0.06, 0.2) | 5.96 (3.09, 10.07) |
| 45 to 49 | 781.46 (496.05, 1095.14) | 25343.71 (19711.15, 31919.16) | 0.48 (0.33, 0.67) | 20.83 (14.12, 29.18) | 781.1 (495.6, 1094.76) | 25343.1 (19710.54, 31918.53) | 0.21 (0.11, 0.37) | 9.13 (4.7, 16.39) |
| 50 to 54 | 807.61 (510.24, 1223.38) | 28127.32 (21491.06, 35355.47) | 0.84 (0.58, 1.19) | 32.55 (22.4, 45.79) | 806.99 (509.74, 1222.81) | 28126.32 (21490, 35354.59) | 0.37 (0.19, 0.63) | 14.34 (7.56, 24.18) |
| 55 to 59 | 859.86 (570.2, 1247.63) | 30887.76 (23894.03, 38922.53) | 1.33 (0.93, 1.93) | 45.12 (31.75, 65.68) | 858.93 (569.13, 1246.8) | 30886.38 (23892.44, 38921.26) | 0.56 (0.29, 1) | 19.1 (9.89, 34.57) |
| 60 to 64 | 863.47 (561.3, 1192.87) | 32753.66 (24529.55, 41937.38) | 2.22 (1.6, 3.16) | 64.72 (46.93, 92.26) | 861.87 (559.96, 1191.1) | 32751.45 (24527.13, 41935.43) | 0.86 (0.44, 1.54) | 25.17 (12.84, 45.27) |
| 65 to 69 | 832.02 (530, 1165.14) | 36709.96 (29091.13, 45962.44) | 3.35 (2.39, 4.54) | 82.55 (58.75, 111.94) | 829.72 (527.74, 1163.4) | 36707.07 (29088.39, 45958.75) | 1.26 (0.74, 2.14) | 31.32 (18.29, 53.01) |
| 70 to 74 | 683.25 (437.41, 952.59) | 38516.15 (30317.18, 47308.29) | 4.73 (3.33, 6.38) | 96.21 (67.58, 129.93) | 680.27 (434.58, 949.59) | 38512.44 (30313.98, 47304.42) | 1.92 (1.07, 3.17) | 39.42 (22.15, 64.99) |
| 75 to 79 | 551.45 (337.99, 805.31) | 39478.7 (31289.97, 48510.28) | 7.03 (4.89, 9.71) | 113.84 (79.09, 157.29) | 547.23 (333.92, 800.45) | 39473.67 (31284.64, 48505.71) | 2.85 (1.39, 4.93) | 46.42 (22.86, 80.47) |
| 80 to 84 | 465.11 (296.79, 720.75) | 35183.18 (27337.31, 43768.28) | 10.48 (6.91, 14.38) | 132.86 (88, 183.19) | 459.38 (290.58, 715.24) | 35176.51 (27332.02, 43761.51) | 4.44 (2.31, 7.31) | 56.51 (29.5, 92.79) |
| 85 to 89 | 434.12 (284.65, 646.85) | 28464.16 (21863.83, 36412.43) | 14.09 (9.35, 19.46) | 142.24 (94.97, 195.82) | 426.43 (278.13, 637.99) | 28454.95 (21853.11, 36400.64) | 6.88 (3.92, 11.06) | 69.32 (39.85, 110.94) |
| 90 to 94 | 448.99 (277.23, 650.47) | 23981.48 (18145.06, 31142) | 17.86 (11.16, 26.03) | 157.34 (99.07, 228.23) | 442.79 (272.7, 643.59) | 23977.1 (18141.23, 31136.37) | 9.62 (5.05, 16.71) | 85.04 (45.1, 147.41) |
| 95 plus | 488.01 (270.09, 780.2) | 22083.27 (15672.41, 29701.63) | 21.21 (11.15, 34.69) | 171.14 (90.5, 278.65) | 482.9 (264.6, 772.27) | 22080.64 (15669.92, 29699.66) | 13.52 (5.9, 25.64) | 109.37 (47.94, 207.1) |
| **Male** | | | | | | | | |
| 15 to 19 | 1215.82 (955.28, 1512.81) | 5794.34 (4420.83, 7417.26) | 0.02 (0.01, 0.03) | 1.38 (0.89, 2) | 1215.8 (955.27, 1512.8) | 5794.32 (4420.8, 7417.24) | 0.01 (0, 0.01) | 0.56 (0.17, 1.14) |
| 20 to 24 | 1196.49 (902.72, 1496.28) | 10357.74 (7688.94, 13363.15) | 0.03 (0.02, 0.05) | 2.18 (1.47, 3.13) | 1196.47 (902.69, 1496.26) | 10357.71 (7688.9, 13363.11) | 0.01 (0.01, 0.03) | 1.04 (0.52, 1.85) |
| 25 to 29 | 944.88 (632.59, 1289.58) | 14892.35 (11367.01, 18731.02) | 0.07 (0.04, 0.1) | 4.26 (2.75, 6.49) | 944.83 (632.55, 1289.55) | 14892.23 (11366.88, 18730.91) | 0.03 (0.01, 0.06) | 2.01 (0.85, 3.9) |
| 30 to 34 | 815.38 (560.95, 1129.29) | 18755.14 (14359.52, 23562.04) | 0.15 (0.1, 0.21) | 8.56 (5.72, 12.26) | 815.27 (560.87, 1129.2) | 18754.91 (14359.32, 23561.82) | 0.07 (0.03, 0.12) | 3.95 (1.95, 6.78) |
| 35 to 39 | 703.41 (470.35, 1013.85) | 20788.07 (15848.67, 26812.31) | 0.29 (0.18, 0.43) | 15.24 (9.87, 22.8) | 703.2 (470.11, 1013.64) | 20787.69 (15848.33, 26811.96) | 0.13 (0.05, 0.24) | 7.12 (2.74, 13.03) |
| 40 to 44 | 612.6 (410.03, 873.96) | 23145.92 (18043.48, 29261.99) | 0.56 (0.37, 0.82) | 26.97 (17.98, 39.66) | 612.2 (409.59, 873.54) | 23145.31 (18043, 29261.39) | 0.27 (0.13, 0.47) | 12.89 (6.48, 22.48) |
| 45 to 49 | 560.9 (362.18, 797.02) | 25809.26 (20234.87, 32515.82) | 0.96 (0.66, 1.39) | 41.74 (28.73, 60.35) | 560.21 (361.41, 796.38) | 25808.17 (20233.69, 32514.79) | 0.44 (0.22, 0.79) | 19.27 (9.42, 34.34) |
| 50 to 54 | 551.83 (344.57, 852.14) | 26952.62 (20688.12, 33391.56) | 1.47 (0.97, 2.2) | 56.79 (37.43, 84.83) | 550.76 (343.49, 851.03) | 26950.97 (20686.59, 33389.92) | 0.65 (0.33, 1.15) | 25.22 (12.62, 44.59) |
| 55 to 59 | 581.46 (380.61, 865.5) | 28112.2 (21989.65, 35141.61) | 2.01 (1.31, 2.92) | 68.17 (44.32, 99.18) | 579.99 (379.35, 863.7) | 28110.02 (21987.56, 35139.86) | 0.8 (0.42, 1.45) | 27.3 (14.39, 49.26) |
| 60 to 64 | 608.9 (397.61, 860.75) | 29001.75 (21814.75, 36953.86) | 2.9 (1.94, 4.15) | 84.63 (56.68, 120.83) | 606.65 (394.8, 858.16) | 28998.51 (21811.71, 36951.09) | 1 (0.52, 1.87) | 29.26 (15.35, 54.75) |
| 65 to 69 | 613.71 (384.12, 876.61) | 30998.79 (24366.95, 38673.83) | 3.78 (2.53, 5.21) | 93.01 (62.27, 127.9) | 610.89 (381.48, 874.07) | 30994.95 (24363.29, 38669.82) | 1.28 (0.73, 2.12) | 31.56 (17.92, 52.31) |
| 70 to 74 | 545.63 (350.32, 763.76) | 33082.02 (26138, 40710.66) | 4.85 (3.39, 6.68) | 98.4 (68.9, 135.39) | 542.18 (347.23, 759.36) | 33077.73 (26133.47, 40706.12) | 1.7 (0.97, 2.69) | 34.61 (19.65, 54.75) |
| 75 to 79 | 465.92 (282.9, 682.97) | 35831.2 (28424.02, 44001.15) | 6.88 (4.74, 9.72) | 111.44 (76.9, 157.17) | 461.3 (278.63, 678.25) | 35825.5 (28417.91, 43996.63) | 2.39 (1.15, 4.08) | 38.76 (18.67, 66.09) |
| 80 to 84 | 417.13 (268.24, 651.1) | 36318.45 (28533.16, 44402.61) | 10.05 (7, 13.95) | 127.58 (89.05, 176.68) | 410.73 (260.77, 645.99) | 36310.73 (28526.63, 44395.01) | 3.35 (1.71, 5.54) | 42.54 (21.73, 70.5) |
| 85 to 89 | 377.9 (248.79, 565.24) | 31019.96 (24336.46, 38960.78) | 15.16 (11.14, 20.18) | 153.12 (112.74, 203) | 368.59 (238.82, 556.09) | 31008.63 (24324.54, 38952.09) | 6.12 (3.58, 9.93) | 61.44 (35.99, 99.48) |
| 90 to 94 | 371.89 (229.55, 544.13) | 26650.95 (20731.92, 33809.98) | 19.23 (13.42, 26.87) | 169.03 (117.87, 235.64) | 363.64 (222.61, 535.83) | 26644.42 (20725.14, 33802.54) | 7.97 (4.35, 14.36) | 69.93 (38.32, 126.25) |
| 95 plus | 379.31 (210.59, 596.71) | 24921.12 (18318.08, 32715.57) | 15.42 (9.21, 23.81) | 124.88 (74.99, 193.47) | 374.54 (206.34, 590.75) | 24918.61 (18316.3, 32713.41) | 8.03 (3.29, 15.99) | 65.02 (26.98, 128.91) |

**Table S12. Predicted Burdens of total and non-malignant MASLD based on Bayesian age-period-cohort model in Japan, China, Philippines and Viet Nam in 2021 and 2050.**

|  | **Total MASLD** | | | | **Non-malignant MASLD** | | | |
| --- | --- | --- | --- | --- | --- | --- | --- | --- |
|  | **Japan** | **China** | **Philippines** | **Viet Nam** | **Japan** | **China** | **Philippines** | **Viet Nam** |
| **Absolute burden in 2050** | | | | | | | | |
| **Incident cases(95% UI)** | 434406 (123185, 745627) | 13313290 (485288, 26385117) | 976662 (389503, 1563821) | 503333 (62205, 944461) | 431789 (121309, 742268) | 13316996 (535682, 26332616) | 975980 (388582, 1563378) | 501335 (60489, 942182) |
| **Prevalent cases(95% UI)** | 17938245 (5936211, 29940279) | 489601872 (0, 1240285342) | 23573306 (11390062, 35756550) | 18490385 (8997217, 27983554) | 17936226 (5936278, 29936175) | 489058949 (0, 1236201888) | 23572130 (11390708, 35753552) | 18487639 (8999000, 27976279) |
| **Deaths(95% UI)** | 2179 (0, 5761) | 27308 (0, 101947) | 2278 (0, 5546) | 4028 (0, 9430) | 2072 (0, 10722) | 16162 (0, 48958) | 1169 (0, 3133) | 1865 (0, 4910) |
| **DALYs(95% UI)** | 41545 (0, 106918) | 825463 (0, 4345415) | 54647 (0, 110872) | 96067 (0, 209425) | 51581 (0, 311126) | 321992 (0, 906464) | 31361 (0, 64902) | 34193 (461, 68066) |
| **YLLs(95% UI)** | 39768 (0, 102344) | 811810 (0, 4325019) | 54156 (0, 109873) | 94843 (0, 206839) | 50794 (0, 322376) | 314202 (0, 896067) | 30900 (0, 63954) | 33437 (507, 66501) |
| **YLDs(95% UI)** | 1081 (0, 2902) | 18597 (0, 85114) | 575 (0, 1819) | 1341 (0, 3890) | 816 (0, 2358) | 41433 (0, 437614) | 339 (0, 1330) | 1002 (0, 4102) |
| **Relative burden in 2050** | | | | | | | | |
| **ASIR per 100,000(95% UI)** | 470.21 (144.83, 795.6) | 1213.46 (128.22, 2298.71) | 813.77 (353.47, 1274.06) | 719.58 (717.67, 721.49) | 468.47 (143.22, 793.72) | 1216.49 (132.97, 2300.01) | 813.18 (352.72, 1273.63) | 536.46 (101.26, 971.65) |
| **ASPR per 100,000(95% UI)** | 13197.84 (4659.1, 21736.58) | 26086.72 (-7893.31, 60066.75) | 19192.46 (9632.84, 28752.08) | 18876.19 (18866.4, 18885.99) | 13197.61 (4659.53, 21735.68) | 26094.24 (-7789.62, 59978.1) | 19191.66 (9633.34, 28749.98) | 16143.65 (8081.63, 24205.66) |
| **ASDR per 100,000(95% UI)** | 0.55 (-0.11, 1.21) | 0.62 (-0.46, 1.69) | 1.58 (-0.23, 3.4) | 2.26 (2.18, 2.34) | 0.33 (-0.34, 0.99) | 0.44 (-0.2, 1.07) | 0.78 (-0.2, 1.77) | 0.92 (-0.22, 2.06) |
| **ASR of DALYs (95% UI)** | 15.32 (-3.34, 33.99) | 18.73 (-21.1, 58.56) | 39.98 (3.07, 76.9) | 54.81 (54.3, 55.32) | 11.26 (-15.27, 37.79) | 11.22 (-3.91, 26.35) | 22.9 (1.3, 44.5) | 21.91 (2.2, 41.61) |
| **ASR of YLLs (95% UI)** | 14.54 (-3.19, 32.27) | 18.24 (-20.77, 57.25) | 39.62 (3.05, 76.2) | 54.28 (53.77, 54.79) | 10.5 (-14.82, 35.83) | 10.76 (-3.94, 25.46) | 22.56 (1.27, 43.85) | 21.41 (2.18, 40.64) |
| **ASR of YLDs (95% UI)** | 0.4 (-0.09, 0.9) | 0.46 (-0.45, 1.37) | 0.35 (-0.14, 0.85) | 0.53 (0.5, 0.57) | 0.32 (-0.11, 0.74) | 0.59 (-1.08, 2.26) | 0.19 (-0.11, 0.49) | 0.48 (-0.34, 1.3) |
| **Absolute burden in 2021** | | | | | | | | |
| **Incident cases(95% UI)** | 434406 (123185, 745627) | 13313290 (485288, 26385117) | 976662 (389503, 1563821) | 503333 (62205, 944461) | 431789 (121309, 742268) | 13316996 (535682, 26332616) | 975980 (388582, 1563378) | 501335 (60489, 942182) |
| **Prevalent cases(95% UI)** | 17938245 (5936211, 29940279) | 489601872 (0, 1240285342) | 23573306 (11390062, 35756550) | 18490385 (8997217, 27983554) | 17936226 (5936278, 29936175) | 489058949 (0, 1236201888) | 23572130 (11390708, 35753552) | 18487639 (8999000, 27976279) |
| **Deaths(95% UI)** | 2179 (0, 5761) | 27308 (0, 101947) | 2278 (0, 5546) | 4028 (0, 9430) | 2072 (0, 10722) | 16162 (0, 48958) | 1169 (0, 3133) | 1865 (0, 4910) |
| **DALYs(95% UI)** | 41545 (0, 106918) | 825463 (0, 4345415) | 54647 (0, 110872) | 96067 (0, 209425) | 51581 (0, 311126) | 321992 (0, 906464) | 31361 (0, 64902) | 34193 (461, 68066) |
| **YLLs(95% UI)** | 39768 (0, 102344) | 811810 (0, 4325019) | 54156 (0, 109873) | 94843 (0, 206839) | 50794 (0, 322376) | 314202 (0, 896067) | 30900 (0, 63954) | 33437 (507, 66501) |
| **YLDs(95% UI)** | 1081 (0, 2902) | 18597 (0, 85114) | 575 (0, 1819) | 1341 (0, 3890) | 816 (0, 2358) | 41433 (0, 437614) | 339 (0, 1330) | 1002 (0, 4102) |
| **Relative burden in 2021** | | | | | | | | |
| **ASIR per 100,000(95% UI)** | 470.21 (144.83, 795.6) | 1213.46 (128.22, 2298.71) | 813.77 (353.47, 1274.06) | 719.58 (717.67, 721.49) | 468.47 (143.22, 793.72) | 1216.49 (132.97, 2300.01) | 813.18 (352.72, 1273.63) | 536.46 (101.26, 971.65) |
| **ASPR per 100,000(95% UI)** | 13197.84 (4659.1, 21736.58) | 26086.72 (-7893.31, 60066.75) | 19192.46 (9632.84, 28752.08) | 18876.19 (18866.4, 18885.99) | 13197.61 (4659.53, 21735.68) | 26094.24 (-7789.62, 59978.1) | 19191.66 (9633.34, 28749.98) | 16143.65 (8081.63, 24205.66) |
| **ASDR per 100,000(95% UI)** | 0.55 (-0.11, 1.21) | 0.62 (-0.46, 1.69) | 1.58 (-0.23, 3.4) | 2.26 (2.18, 2.34) | 0.33 (-0.34, 0.99) | 0.44 (-0.2, 1.07) | 0.78 (-0.2, 1.77) | 0.92 (-0.22, 2.06) |
| **ASR of DALYs (95% UI)** | 15.32 (-3.34, 33.99) | 18.73 (-21.1, 58.56) | 39.98 (3.07, 76.9) | 54.81 (54.3, 55.32) | 11.26 (-15.27, 37.79) | 11.22 (-3.91, 26.35) | 22.9 (1.3, 44.5) | 21.91 (2.2, 41.61) |
| **ASR of YLLs (95% UI)** | 14.54 (-3.19, 32.27) | 18.24 (-20.77, 57.25) | 39.62 (3.05, 76.2) | 54.28 (53.77, 54.79) | 10.5 (-14.82, 35.83) | 10.76 (-3.94, 25.46) | 22.56 (1.27, 43.85) | 21.41 (2.18, 40.64) |
| **ASR of YLDs (95% UI)** | 0.4 (-0.09, 0.9) | 0.46 (-0.45, 1.37) | 0.35 (-0.14, 0.85) | 0.53 (0.5, 0.57) | 0.32 (-0.11, 0.74) | 0.59 (-1.08, 2.26) | 0.19 (-0.11, 0.49) | 0.48 (-0.34, 1.3) |

**Figure S1. Years lived with disability (YLDs) and Years of life lost (YLLs) of total MASLD burden, by countries, years and ages, respectively, in the Western Pacific region in 1990–2021.**


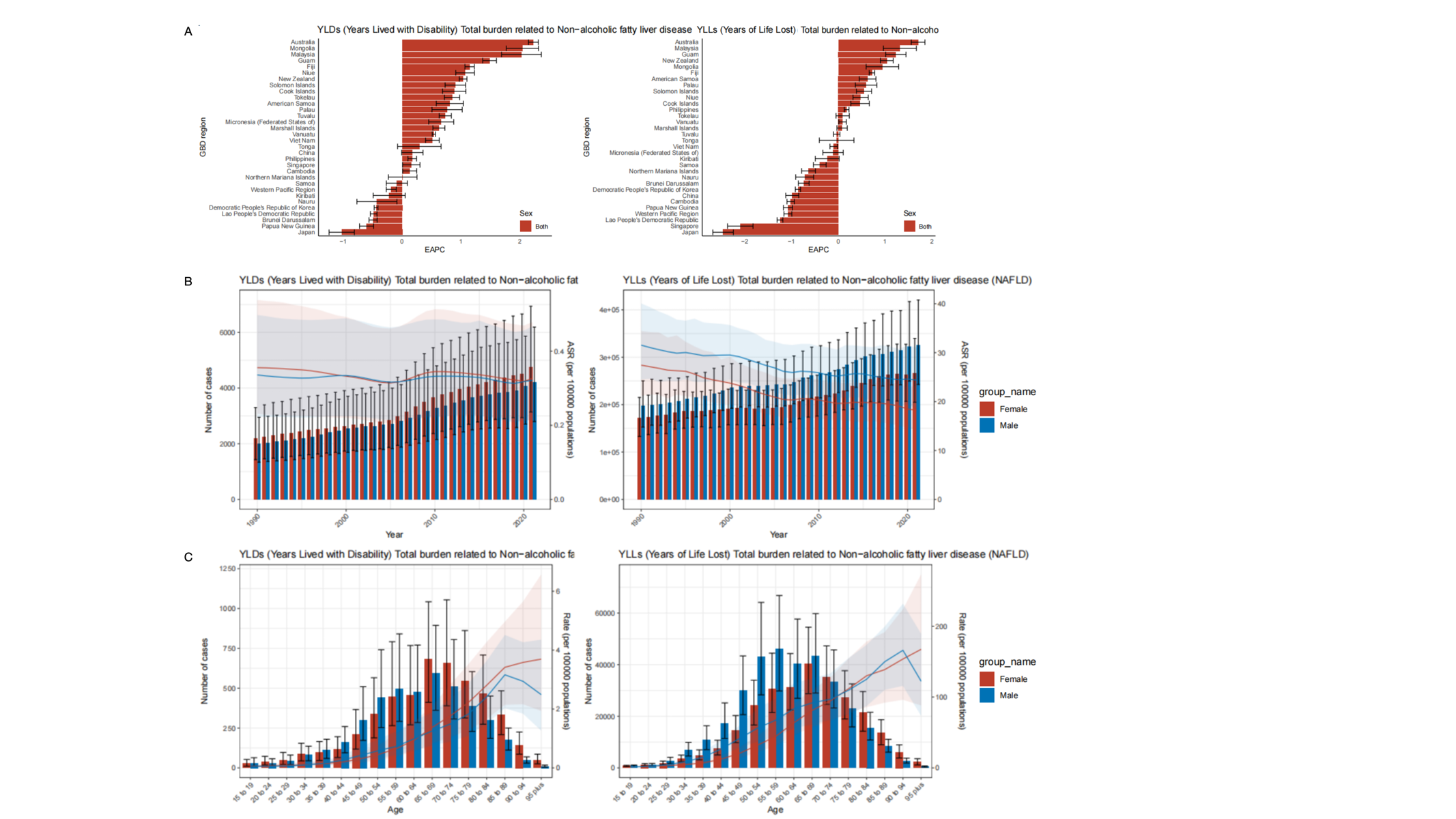


**Figure S2. Years lived with disability (YLDs) and Years of life lost (YLLs) of non-malignant MASLD burden, by countries, years and ages, respectively, in the Western Pacific region in 1990–2021.**


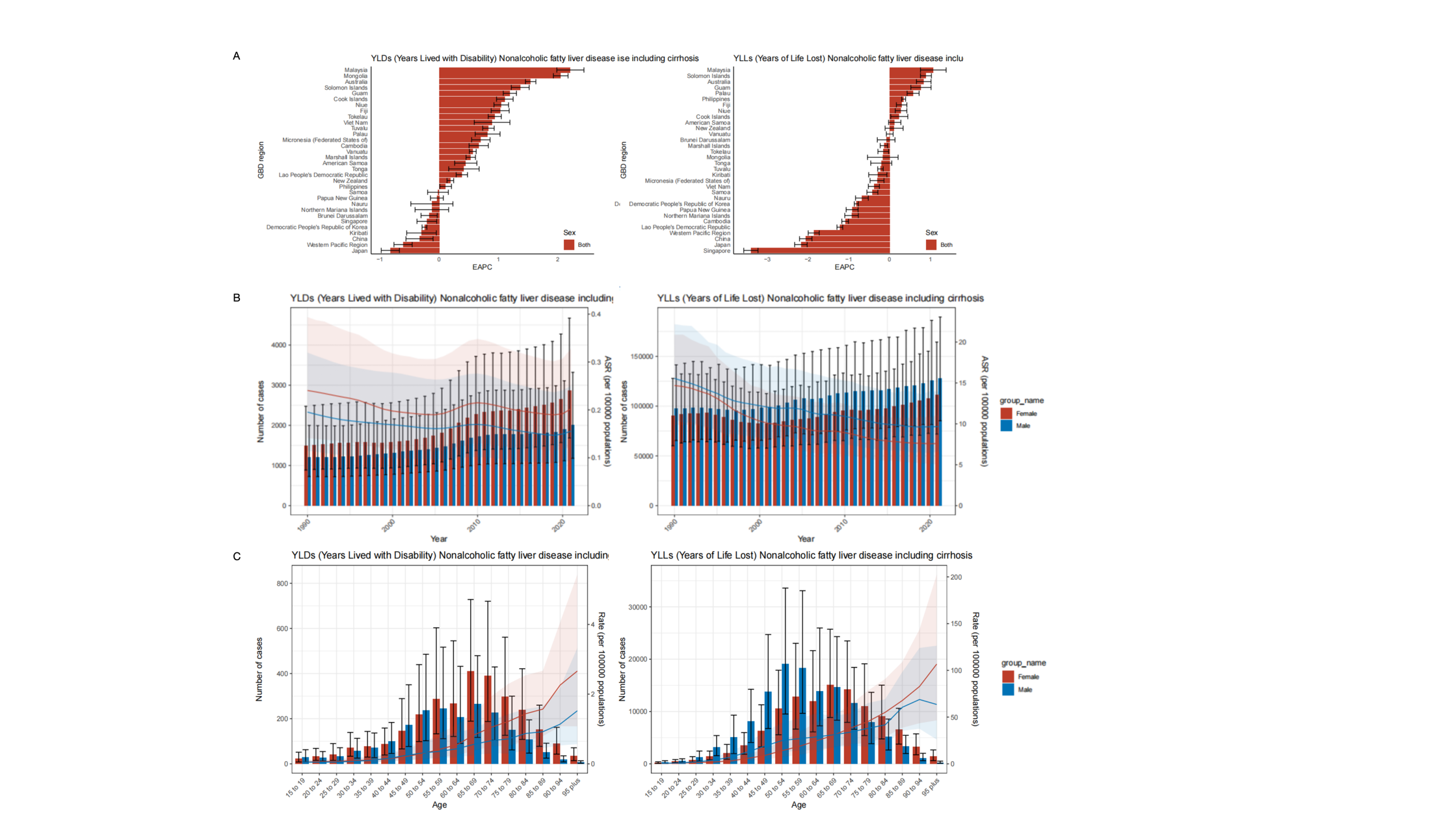

Supplement: Supplementary file 1 — Appendix S1. [file FSN3-13-e70627-s001.docx]
